# ARPC1B promotes mesenchymal phenotype maintenance and radiotherapy resistance by blocking TRIM21-mediated degradation of IFI16 and HuR in glioma stem cells

**DOI:** 10.1186/s13046-022-02526-8

**Published:** 2022-11-16

**Authors:** Zijie Gao, Jianye Xu, Yang Fan, Zongpu Zhang, Huizhi Wang, Mingyu Qian, Ping Zhang, Lin Deng, Jie Shen, Hao Xue, Rongrong Zhao, Teng Zhou, Xing Guo, Gang Li

**Affiliations:** 1 Department of Neurosurgery, Qilu Hospital, Cheeloo College of Medicine and Institute of Brain and Brain-Inspired Science, Shandong University, Jinan, 250012 Shandong China; 2 Shandong Key Laboratory of Brain Function Remodeling, Jinan, 250012 Shandong China; 3 Key Laboratory of Post-Neuroinjury Neuro-Repair and Regeneration in Central Nervous System, Ministry of Education and Tianjin City, Tianjin Neurological Institute, Tianjin Medical University General Hospital, Tianjin, 300052 China; 4 Department of Thoracic Head Neck Medical Oncology, The University of Texas MD Anderson Cancer Center, Houston, TX 77030 USA

**Keywords:** Glioblastoma, Glioma stem cells, ARPC1B, Radiotherapy resistance, AZD6738

## Abstract

**Background:**

Intratumoral heterogeneity is the primary challenge in the treatment of glioblastoma (GBM). The presence of glioma stem cells (GSCs) and their conversion between different molecular phenotypes contribute to the complexity of heterogeneity, culminating in preferential resistance to radiotherapy. ARP2/3 (actin-related protein-2/3) complexes (ARPs) are associated with cancer migration, invasion and differentiation, while the implications of ARPs in the phenotype and resistance to radiotherapy of GSCs remain unclear.

**Methods:**

We screened the expression of ARPs in TCGA-GBM and CGGA-GBM databases. Tumor sphere formation assays and limiting dilution assays were applied to assess the implications of ARPC1B in tumorigenesis. Apoptosis, comet, γ-H2AX immunofluorescence (IF), and cell cycle distribution assays were used to evaluate the effect of ARPC1B on radiotherapy resistance. Immunoprecipitation (IP) and mass spectrometry analysis were used to detect ARPC1B-interacting proteins. Immune blot assays were performed to evaluate protein ubiquitination, and deletion mutant constructs were designed to determine the binding sites of protein interactions. The Spearman correlation algorithm was performed to screen for drugs that indicated cell sensitivity by the expression of ARPC1B. An intracranial xenograft GSC mouse model was used to investigate the role of ARPC1B in vivo.

**Results:**

We concluded that ARPC1B was significantly upregulated in MES-GBM/GSCs and was correlated with a poor prognosis. Both in vitro and in vivo assays indicated that knockdown of ARPC1B in MES-GSCs reduced tumorigenicity and resistance to IR treatment, whereas overexpression of ARPC1B in PN-GSCs exhibited the opposite effects. Mechanistically, ARPC1B interacted with IFI16 and HuR to maintain protein stability. In detail, the Pyrin of IFI16 and RRM2 of HuR were implicated in binding to ARPC1B, which counteracted TRIM21-mediated degradation of ubiquitination to IFI16 and HuR. Additionally, the function of ARPC1B was dependent on IFI16-induced activation of NF-κB pathway and HuR-induced activation of STAT3 pathway. Finally, we screened AZD6738, an ataxia telangiectasia mutated and rad3-related (ATR) inhibitor, based on the expression of ARPC1B. In addition to ARPC1B expression reflecting cellular sensitivity to AZD6738, the combination of AZD6738 and radiotherapy exhibited potent antitumor effects both in vitro and in vivo.

**Conclusion:**

ARPC1B promoted MES phenotype maintenance and radiotherapy resistance by inhibiting TRIM21-mediated degradation of IFI16 and HuR, thereby activating the NF-κB and STAT3 signaling pathways, respectively. AZD6738, identified based on ARPC1B expression, exhibited excellent anti-GSC activity in combination with radiotherapy.

**Supplementary Information:**

The online version contains supplementary material available at 10.1186/s13046-022-02526-8.

## Background

Glioblastoma (GBM), characterized by wild-type isocitrate dehydrogenase (IDH), is the most aggressive and fatal primary brain tumor in adults [1, 2]. Despite substantial efforts, the standard of care for GBM, maximum surgical resection followed by ionizing radiation (IR) and temozolomide adjuvant therapy, has only a minor clinical benefit [3, 4]. Due to the transcriptional plasticity and heterogeneity of GBM, radiotherapy resistance and eventual recurrence are inevitable [5].

According to bulk expression profiles, there are three phenotypes of GBM, termed proneural (PN), classical (CL) and mesenchymal (MES). GBM patients with the MES phenotype exhibit worse survival and enhanced resistance to radiotherapy than patients with the PN phenotype [6, 7]. Studies have reported that during the natural evolution of GBM or in response to radiotherapy, a PN-to-MES transition (PMT) occurs [8, 9]. The existence of glioma stem cells (GSCs) is a key factor driving PMT and the heterogeneity of GBM [10].

GSCs are characterized by multilineage differentiation and self-renewal ability, exhibiting preferential resistance to radiotherapy and chemotherapy [11, 12]. Similar to the transformation of GBM, PN GSCs have a tendency to transform into MES GSCs to resist the adverse effects of treatment [13, 14]. Previous studies have identified STAT3 and C/EBPβ as two master regulators (MRs) of PMT [15]. Besides, PMT process is also implicated in TAZ, the NF-κB signaling pathway and the tumor microenvironment (TME) [16–18]. We also recently reported that macrophage-derived small extracellular vesicles (sEVs) were key regulators of PMT in GSCs [19] and that cell surface GRP78 was upregulated in MES GSCs and played a pivotal role in the maintenance of the MES phenotype [20]. Despite considerable studies exploring the evolution of PMT, the intrinsic molecular mechanism of PMT in GSCs remains elusive.

The actin-related protein-2/3 (ARP2/3) complex was the first molecule identified to initiate new filament polymerization [21]. It is an assembly of seven polypeptides, including ARP2, ARP3, and five scaffolding subunits, ARPC1 to ARPC5. Unlike the other four ARPC subunits, ARPC1 is a WD-repeat-containing protein consisting of two isoforms, called ARPC1A and ARPC1B [22]. Extensive studies have demonstrated that ARPs are dysregulated in cancer. In general, ARPs have numerous crucial biological functions, involving regulation of cell differentiation, migration, adhesion, as well as cargo transport [22, 23]. Recent studies have demonstrated that ARPs are associated with malignant behavior in colorectal, breast and lung cancers and that they act in promoting cancer cell invasion and metastasis [24–29]. Liu et al. recently discovered that ARPC1B in macrophages promoted motility and epithelial-mesenchymal transition (EMT) of glioma cells [30]. Other studies have revealed that ARPC1B is a risk factor for predicting survival in GBM and uveal melanoma [31, 32]. Currently, the effects and detailed mechanisms of ARPC1B in GBM phenotypes and radiotherapy are unknown.

The main strategy for treatment of refractory or recurrent gliomas is the combination of standard radiotherapy and chemotherapy with targeted agents. Ataxia telangiectasia and Rad3-related (ATR), serving as a serine/threonine protein kinase, plays an essential role in coordinating the DNA damage response and maintaining genomic stability [33]. A growing number of studies reported that targeting ATR alone or in combination with other treatment modalities exhibited excellent anti-tumor effect [34]. Here, we performed drug sensitivity analysis and found that the expression of ARPC1B is reflective of sensitivity to AZD6738, which is a selective ATR inhibitor. AZD6738 has been widely evaluated the efficiency among colorectal cancer, head and neck cancer, lung cancer, neuroblastoma, etc. [35–39]. Besides, AZD6738 exhibited a promising antitumor activity in a phase II study for the treatment of advanced melanoma [40]. However, the study of AZD6738 on GBM or GSC cells is limited.

In this study, we found that ARPC1B was upregulated in the MES phenotype and predicted a poor outcome. In vitro and in vivo studies demonstrated that knockdown of ARPC1B suppressed the MES phenotype and promoted radiotherapy sensitivity in GSCs, whereas overexpression of ARPC1B promoted MES phenotype transformation and radiotherapy resistance. Mechanistically, ARPC1B directly binds to IFI16 and HuR, preventing TRIM21-mediated degradation of IFI16 and HuR by ubiquitination, thereby activating the STAT3 and NF-κB signaling pathways, respectively. As discussed previously, activation of STAT3 and NF-κB facilitated conversion of PN-GSCs to MES-GSCs, which exhibited greater resistance to radiotherapy. Moreover, we identified a selective ATR inhibitor, AZD6738, based on the expression of ARPC1B. We validated that AZD6738 in combination with radiotherapy exhibited excellent anti-GSC activity for the first time.

## Materials and methods

### GSCs and culture

The patient-derived PN-GSCs (GSC 8-11 and GSC 11), MES-GSCs (GSC 20, GSC 267 and GSC 28) and neural progenitor cell (NPC) were a kind gift from Dr. Frederick F. Lang and Dr. Krishna P.L. Bhat (M.D. Anderson Cancer Center, University of Texas, Houston, TX). Phenotypes of GSCs in this study have been identified according to the genetic signature of Philips and Verhaak, and are widely accepted and applied [8, 16, 17, 41]. GSCs and NPC were digested into single cells by Accutase solution (Sigma-Aldrich, USA) and then cultured in DMEM/F12 (Gibco, USA) supplemented with B-27 (Gibco, USA), 20 ng/mL recombinant human (rh) epidermal growth factor (R&D Systems, USA) and 20 ng/mL rh basic fibroblast growth factor (R&D Systems, USA). The cells were cultured at 37 °C in a humid chamber with 5% carbon dioxide and 5% oxygen.

### Lentiviral transfection

We performed persistent knockdown of ARPC1B by lentivirus expressing sh-ARPC1B, which were synthesized by GeneChem (Shanghai, China). The ARPC1B, IFI16 and HuR overexpression and the corresponding control lentiviruses were also synthesized by GeneChem (Shanghai, China). The RNA-interfering sequences used in this study are listed in supplementary materials and methods.

### Real-time quantitative RT-PCR (qRT-PCR)

The total RNA was extracted by TRIzol (Invitrogen, USA) following manufacturer’s protocol. We performed reverse transcription using high-capacity-cDNA Reverse Transcription Kit (Toyobo, China) according to the protocol. The PCR primer pairs’ sequences were listed in supplementary materials and methods and an Mx-3000P Quantitative PCR System (Applied Biosystems, USA) was leveraged for qRT-PCR.

### Tumor sphere formation assay

GSCs were inoculated into 6-well plates at a density of 1000 cells per well and cultured for 7 days with 1.5 ml of GSC culture medium. The relative diameters of tumor spheres were recorded by an optical microscope and used for subsequent analysis.

### Extreme limiting dilution assay (ELDA)

GSCs were inoculated into 96-well plates at a gradient of 0, 2, 4, 8, 16, 32, 64 or 128 cells per well and with 10 replicate wells per set. We observed and recorded the number of wells with tumor spheres formation 7 days later, and analyzed the collecting data using (http://bioinf.wehi.edu.au/software/elda/).

### IR treatment

GSCs were treated with IR (6 Gy) for in vitro radiotherapy assays. The apoptosis, comet assay, cell-cycle analysis and γ-H2AX IF staining assay were performed 96 h later. In term of mouse experiments, we treated mice with 4 doses of IR (2.5 Gy each) within 7 to 14 days after GSCs injection.

### Apoptosis assay

GSCs were treated with IR (6 Gy) and total GSCs were collected after 96 h to assess apoptotic rate. We leveraged Apoptosis detection kit (BD Biosciences, USA) for apoptosis detection in accordance with manufacturer’ s protocol. The final results were generated by a BD Accuri C6 flow cytometer (BD Biosciences, USA) and further processed by FlowJo V10.

### Alkaline comet assay

We performed alkaline comet assay to detect the DNA damage levels following manufacturer’s instruction [42]. In brief, GSCs with different interventions were collected and harvested in PBS at 1*10^5^ cells/ml density. We then mixed the GSCs with LM agarose in a ratio of 1:10 (V/V) and added 50 µl of the mixture onto a comet slide immediately. We further lysed the cells using alkaline lysis buffer for 12 h at 4℃ and soaked the slides into alkaline electrophoresis solution for 20 min. Then we performed electrophoresis procedure for slides at 25 V for 30 min. Finally, we stained slides with Green-DNA Dye and collected images with a fluorescence microscopy.

### Transferase dUTP nick end labeling (TUNEL) assay

TUNEL assay was conducted using TUNEL apoptosis assay kit (C1090; Beyotime, China) in accordance with manufacturer’s instruction. In brief, tissue sections of mice with different interventions were fixed with 4% paraformaldehyde and washed with PBS. Then the sections were permeabilized in PBS with 0.5% Triton X-10. The detection solution and DAPI solution were prepared for staining apoptotic cells and nuclei, respectively. We captured images with a fluorescent microscope and recorded the percentage of apoptotic cells.

### Cell cycle assay

GSCs were treated with IR (6 Gy), and dissociated and fixed in cold ethanol after 96 h. Then we resuspended and stained the GSCs with propidium iodide (PI) staining solution (BD Biosciences, USA). Finally, we conducted cell-cycle analysis leveraging a a BD Accuri C6 flow cytometer.

### Western blotting (WB), Immunohistochemistry (IHC) and immunofluorescence (IF)

For Western blotting assay, we lysed GSCs in different groups using RIPA with 1% protease and phosphate inhibitor. We then separated proteins by electrophoresis using SDS-PAGE gels and transferred the proteins to polyvinylidene fluoride (PVDF) membrane. Primary anti-bodies were incubated with membranes at 4 °C overnight. After that, we removed primary antibodies and incubated the blots with secondary antibodies at room temperature for 1 h. We examined proteins using an Odyssey fluorescence scanner (ChemiDoc XRSþ, Bio-Rad, USA).

For IHC assay, tissue sections from GSC xenografted mice were stained with the primary antibodies against CD44. We captured images of sections with an optical microscope and evaluated them quantitatively in accordance with German IHC scoring system [43].

For IF assay, we attached and fixed GSCs in μ-slide 8-well plates (bidi, Germany). We then permeabilized and blocked GSCs using 0.3% Triton X-100 as well as 5% Goat serum, respectively. After that, we incubated the GSCs with primary antibodies at 4 °C overnight, and then incubated GSCs with fuorescent secondary antibodies and DAPI for 1 h and 15 min, respectively. GSCs were washed three times with PBS after each staining or process. Finally, the representative images were collected leveraging a LeicaSP8 confocal microscope for further analysis.

All antibodies used in this research are presented in supplementary materials and methods.

### Co-immunoprecipitation (Co-IP)

We conducted co-IP assay leveraging Pierce Classic Magnetic IP/co-IP Kit (Thermo Fisher, USA) in accordance with manufacturer’s protocol. In brief, we incubated antibodies with protein A/G magnetic beads. Then we obtained GSCs lysates and mixed with antibody coupled beads overnight at 4 °C. After washing and denaturation procedures, proteins interacting with the beads are collected for western blotting.

### Cycloheximide (CHX) assay

CHX is a protein translation inhibitor, widely used to analyze half-life of proteins. We treated different groups of GSCs with 100 μg/ml CHX for 0 h, 3 h, 6 h or 9 h, followed by collecting proteins and performing western blotting assay to detect protein levels.

### In vivo studies

The animal experiments were approved by the Institutional Animal Care and Use Committee of Qilu Hospital of Shandong University. We purchased 4-week-old male BALB/c nude mice from the Model Animal Research Center of Nanjing University (Nanjing, China). We randomly grouped similarly situated mice into different groups in preparation for construction of intracranial GSCs xenograft models. We prepared luciferase labeled GSC 267, GSC 20 and GSC 8-11 cells with different interventions of ARPC1B expression. We injected 1 × 10^6^ GSCs into the right frontal lobe of the mice for the construction of GSCs xenografts. For the IR treatment group, mice were given four doses of IR (2.5 Gy each) between 7 and 14 days postoperatively. We assessed in vivo tumor growth by intraperitoneal injection of 150 mg/kg of fluorescein followed by bioluminescence. Representative images and quantification of bioluminescence were obtained from an IVIS Lumina series III ex vivo imaging system (PerkinElmer, USA).

### Public data collection

Transcript level data and associated clinical information of TCGA GBM were extracted from GDC Data Portal (https://portal.gdc.cancer.gov/). The RNA-seq transcriptome data and clinical traits of the CGGA GBM were downloaded from CGGA database (http://www.cgga.org.cn/).

### Single-cell RNA sequencing analysis

The single-cell RNA-sequencing of gliomas were downloaded from Gene Expression Omnibus (GEO, https://www.ncbi.nlm.nih.gov/geo/, GSE138794) and analyzed using R package “Seurat 4.1.0”. Method “UMAP” was applied for the visualization of different cell clusters. R package “irGSEA” was used to calculate and visualize the enrichment scores of the Verhaak_GBM_MES signature by method “UCell”.

### Gene set enrichment analysis (GSEA)

We collected the gene signatures of “Phillips glioblastoma mesenchymal” as well as “Phillips glioblastoma proneural” from the research of Bhat. et. [16]. The GSEA_4.1.0 software was leveraged for running GSEA.

### Association of ARPC1B expression with drug sensitivity

We downloaded the drug sensitivity data of GBM cell lines from Genomics of Drug Sensitivity in Cancer (GDSC, www.cancerRxgene.org) [44], whereas the transcriptional data for corresponding cells were obtained from Cancer Cell Line Encyclopedia (CCLE, https://portals.broadinstitute.org/ccle/). The drugs significantly implicated in the expression of ARPC1B were screened according to Spearman correlation analysis.

### Drugs and drugs dose response curves

We purchased AZD6738 (Synonyms: Ceralasertib) from MedChemExpress (MCE, China). AZD6738 which was dissolved in dimethyl sulfoxide (DMSO) was stored at -20 °C and used up within one month. GSCs were inoculated into 96-well plates at a density of 5000 cells per well, and treated with different concentrations of AZD6738. The GSCs were cultured at 37 °C for 48 h. After that, we added 10 μl Cell Counting Kit-8 (CCK-8) solution to each well and the absorbance reflecting cell proliferation were measured 1 h later. GSCs were treated with 1 μM AZD6738 prior to IR intervention in vitro experiments. For in vivo experiments, AZD6738 was dissolved in a solution containing 10% DMSO, 40% propylene glycol and 50% deionized water, and administered orally to mice at a dose of 50 mg/kg.

### Statistical analysis

We leveraged GraphPad Prism 8.0 software and R 4.1.3 to perform all statistical analysis. The normality of distribution and the homogeneity of variance were proved by Shapiro–Wilk normality test and Bartlett test, respectively. The student’s t-Test and one-way ANOVA were performed to compare the certified data (significance > 0.1) between two groups and more than two groups, respectively. We performed Pearson correlation algorithm to evaluate the correlation between different groups. For survival analysis, Kaplan–Meier (KM) curve and log-rank test were performed to visualize and assess survival between different groups, respectively. *P*-value < 0.05 were accepted as statistically significant (*p-value < 0.05; **p-value < 0.01; ***p-value < 0.001).

## Results

### ARPC1B is significantly associated with the MES phenotype and predicts a poor outcome of GBM

**Fig. 1:**
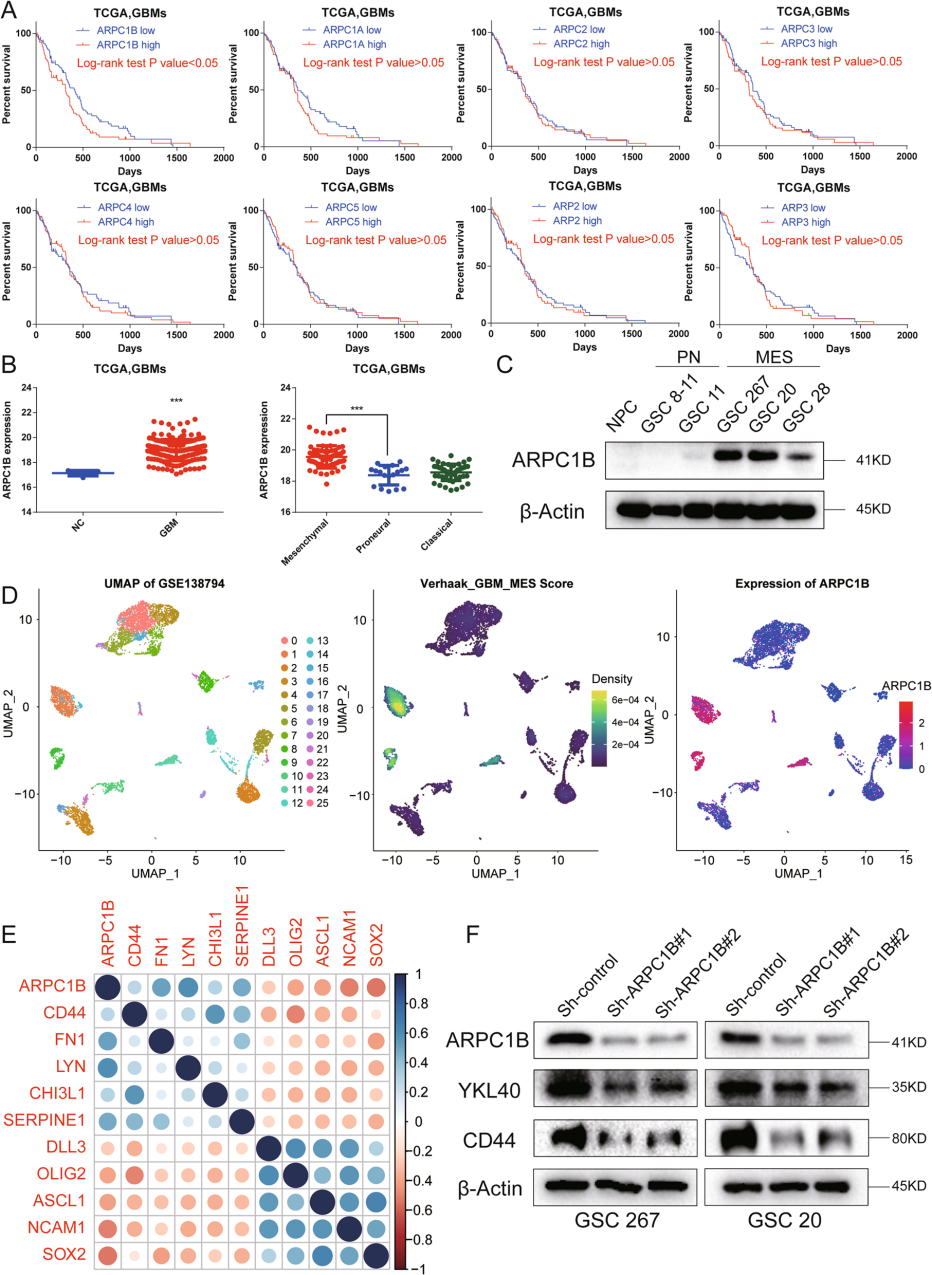
ARPC1B is significantly associated with the MES phenotype and predicts a poor outcome of GBM. **A** Kaplan–Meier curves revealing overall survival of TCGA-GBM patients stratified in accordance with ARPs’ expression. **B** The expression of ARPC1B in normal control and GBM tissues (left panel) and the expression of ARPC1B in mesenchymal (MES), proneural (PN), and classical (CL) phenotypes (right panel) in TCGA GBM. **C** Western blotting of ARPC1B expression in NPCs, MES-GSCs (GSC 20, GSC 267, and GSC 28) and PN-GSCs (GSC 8-11 and GSC 11). **D** Single-cell RNA sequencing of GSE138794 visualizing UMAP cell clusters, Verhaak_GBM_MES score and ARPC1B expression. **E** The correlation of ARPC1B expression with PN-associated (DLL3, OLIG2, ASCL1, NCAM1, and SOX2) and MES-associated genes (CD44, FN1, LYN, CHI3L1, and SERPINE1). **F** Western blot analysis of ARPC1B, CD44 and YKL-40 protein expression upon ARCP1B knockdown in GSCs. β-Actin served as the control

To explore the role of ARPs in GBM phenotypes, we investigated the expression of ARPs and conducted KM survival analysis. We found that in TCGA-GBM, the expression levels of ARPC1B, ARPC2, ARPC3, ARP2, and ARP3 were obviously higher in MES phenotype than in PN phenotype, whereas ARPC1B was a unique molecule correlated with a poor prognosis with a significant *p* value (Fig. 1A, B and Fig. S1A). Similar outcomes were found for CGGA-GBM analysis, where the expression levels of ARPC1B, ARPC3 and ARPC5 were obviously upregulated in the MES phenotype in comparison with the PN phenotype (Fig. S1C). In addition, GBM patients with low expression of ARPC1B survived significantly longer than those with high expression of ARPC1B (*P* value < 0.001, Fig. S1B). Collectively, these data implied that among ARPs, ARPC1B was most associated with the MES phenotype and prognosis of GBM.

We further utilized GSCs considered invaluable for GBM analysis to compare the expression of ARPC1B between different phenotypes of GSCs. The MES GSCs (GSC 267, GSC 20 and GSC 28) exhibited an obviously high expression of ARPC1B, whereas the PN GSCs (GSC 8-11 and GSC 11) and NPC barely expressed ARPC1B (Fig. 1C). GSEA showed that the group with high ARPC1B expression was more enriched in MES phenotype, while the group with low ARPC1B expression was more enriched in PN phenotype (Fig. S1D). Single-cell RNA sequencing analysis showed that the cell clusters with high expression of APRC1B specifically exhibited higher Verhaak_GBM_MES scores, whereas the consistencies between the other ARPs’ expression and Verhaak_GBM_MES scores were not evident (Fig. 1D and Fig. S1E). We performed correlation analysis based on the TCGA and CGGA databases and found that ARPC1B expression was positively correlated with MES phenotype marker genes (CD44, FN1, LYN, CHI3L1, and SERPINE1), whereas it was negatively correlated with PN phenotype marker genes (DLL3, OLIG2, ASCL1, NCAM1, SOX2) (Fig. 1E, Fig. S2A and Table S1). Then, we performed western blotting and revealed that knockdown of ARPC1B inhibited CD44 and YKL-40 expression in GSC 267 and GSC 20 cells, whereas overexpression of ARPC1B inhibited SOX2 expression in GSC 8-11 cells (Fig. 1F and Fig. S2B).

### ARPC1B promotes self-renewal and IR resistance of GSCs in vitro

**Fig. 2:**
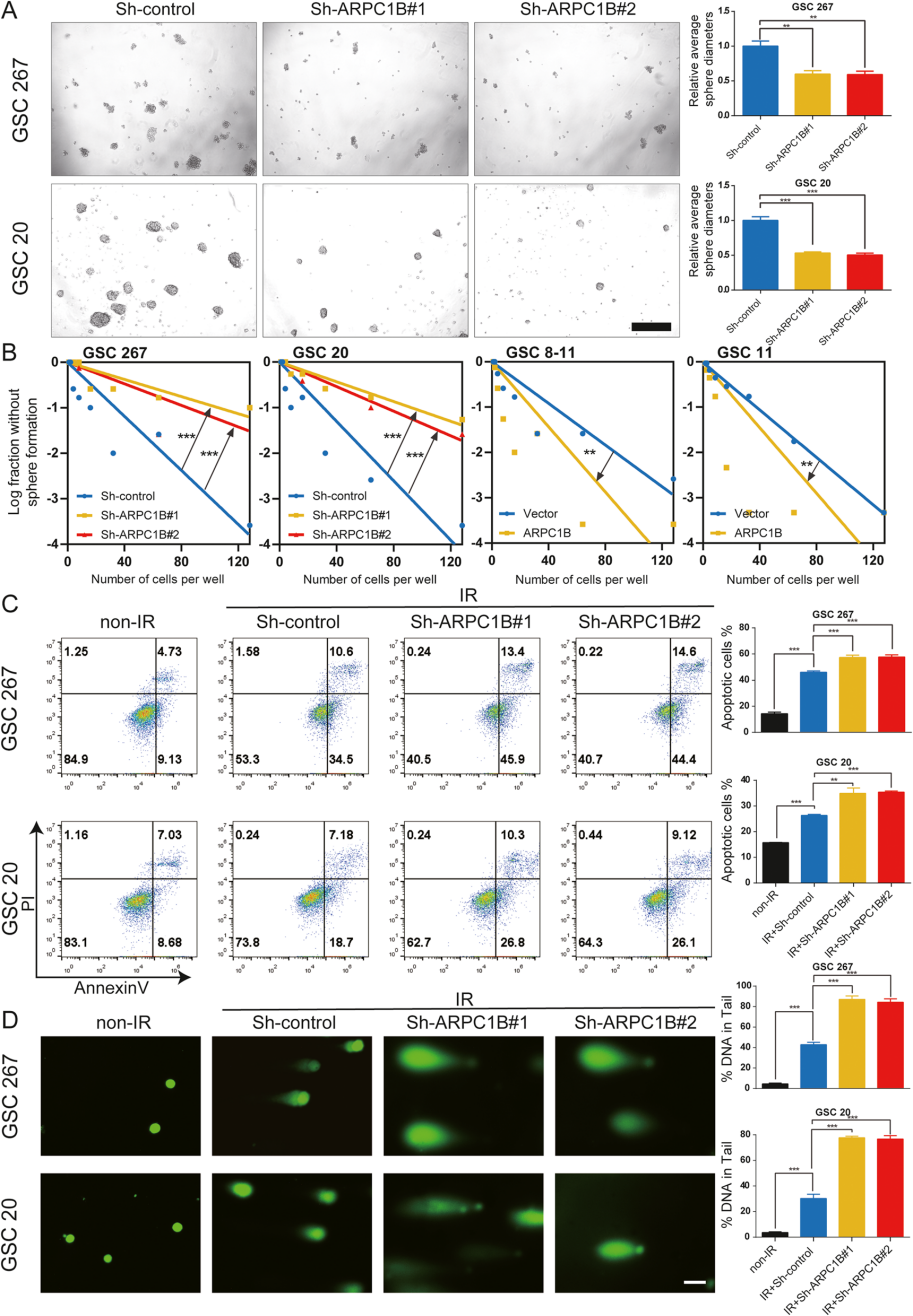
ARPC1B promotes the self-renewal and IR resistance of GSCs in vitro. **A** Representative images and quantification of tumor sphere formation of GSCs transduced with two different sh-ARPC1B sequences. Scale bar, 100 μm. **B** Limiting dilution assays of MES GSCs expressing sh-control or sh-ARPC1B and PN GSCs expressing vector or ARPC1B. **C** Flow cytometric analysis showing the impact of ARPC1B knockdown on the apoptosis of GSCs treated with IR (6 Gy). The right panels show the quantification of the apoptosis rate. **D** Representative images and quantification of comet assays showing the effect of ARPC1B knockdown on DNA damage in GSCs treated with IR (6 Gy). Scale bar, 20 μm

Since ARPC1B is closely associated with GBM phenotypes, we performed a tumor sphere formation assay and limiting dilution assay to evaluate the function of ARPC1B on GSC biology. The results demonstrated that stable knockdown of ARPC1B resulted in decreased sphere diameter and sphere formation ability of GSC 20 and GSC 267, which implied that the self-renewal ability of MES GSCs was inhibited (Fig. 2A, B). Overexpression of ARPC1B increased the sphere diameter and sphere formation ability of GSC 8-11 and GSC 11 cells (Fig. S2C, D and Fig. 2B).

GSCs, especially MES GSCs, show excellent resistance to IR. We employed a series of assays in vitro to assess the role of ARPC1B in IR treatment, including apoptosis, comet, γ-H2AX IF, and cell cycle experiments. The number of apoptotic GSCs was obviously elevated in the ARPC1B knockdown combined with IR treatment group of GSC 20 and GSC 267 (Fig. 2C). In addition, overexpression of ARPC1B enhanced the resistance of GSC 8-11 cells to IR treatment (Fig. S3A). Besides, we found the expression of ARPC1B was upregulated with increasing dose of IR treatment (Fig. S2E).

The response to IR could also be reflected by the percentage of cells arrested in G2/M phase [45]. We performed cell cycle distribution assessment and found that for GSC 20 and GSC 267, the proportion of cells in G2/M phase was significantly higher in the ARPC1B knockdown with IR treatment group (Fig. S3E). For GSC 8-11, overexpression of ARPC1B attenuated the G2/M cell arrest induced by IR intervention (Fig. S3F).

Comet assay and γ-H2AX IF were used to assess DNA damage. We detected a significant increase in DNA damage in the sh-ARPC1B groups after IR treatment using the comet assay, coinciding with the IF results for the radiation injury marker γ-H2AX (Fig. 2D and Fig. S3C). Meanwhile, the degree of DNA damage in GSCs 8-11 caused by radiotherapy was reduced by ARPC1B overexpression (Fig. S3B, D). Altogether, we concluded from these in vitro experiments that ARPC1B not only improved the self-renewal ability of GSCs but also increased the resistance of GSCs to radiotherapy.

### ARPC1B promotes GSC progression and IR resistance in vivo

**Fig. 3:**
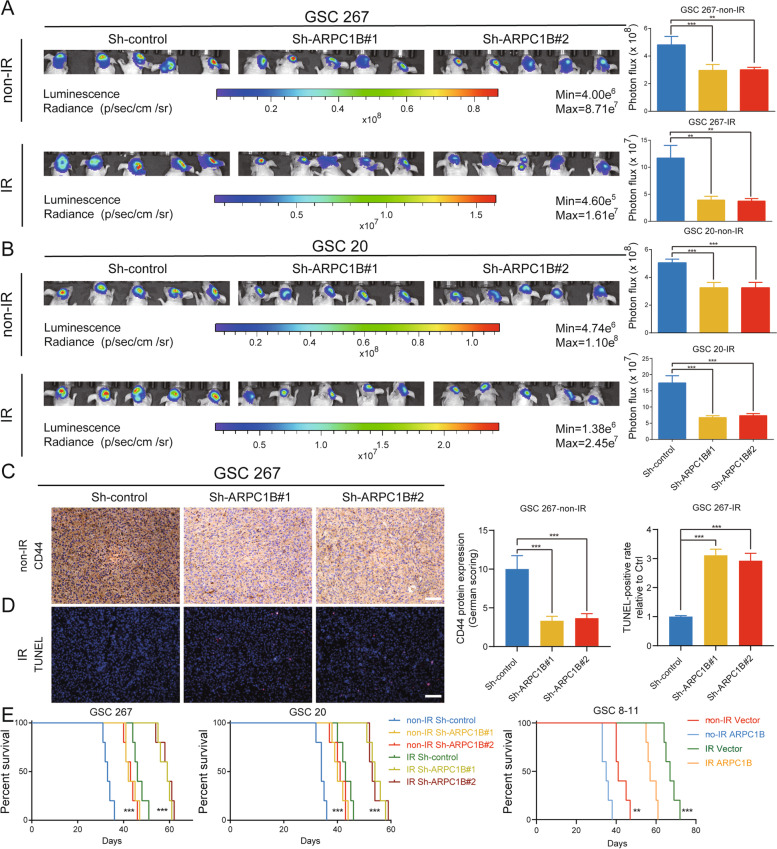
ARPC1B promotes GSC progression and IR resistance in vivo. **A**, **B** Bioluminescence imaging of tumor size on day 30 in sh-control, sh-ARPC1B#1 and sh-ARPC1B#2 GSC 267 (**A**) or GSC 20 (**B**) xenograft nude mice receiving or not receiving IR treatment. The right panel shows the quantification of photon counts of GSC 267 and GSC 20 xenografts. **C** Representative images and quantification of IHC staining for CD44 in sections of non-IR GSC 267 xenografts. Scale bar, 200 μm. **D** Representative images and quantification of TUNEL staining in sections of IR-treated GSC 267 xenografts. Scale bar, 200 μm. **E** Kaplan–Meier curves visualizing the survival of GSC 267, GSC 20 and GSC 8-11 xenograft mice in different groups

To evaluate the function of ARPC1B on the proliferation and radiotherapy resistance of GSCs in an in vivo setting, we constructed xenograft mouse models for in vivo studies. ARPC1B knockdown or control GSCs were implanted in situ into brains of nude mice. Although the tumor burdens were similar in the three groups on Day 7 (Fig. S4A), orthotopic xenografting of GSC 267 with ARPC1B knockdown exhibited a suppression of tumor proliferation (Fig. 3A). For the GSC 267 IR treatment cohort, knockdown of ARPC1B inhibited tumor progression even more after four cycles of 2.5 Gy IR (Fig. 3A). We further validated this finding in GSC 20 cells and obtained similar results (Fig. 3B and Fig. S4B). Additionally, overexpression of ARPC1B promoted tumor progression and resistance to radiotherapy in GSC 8-11 xenograft mice (Fig. S4C, D).

We performed IHC, TUNEL and H&E staining for tumor tissue sections. The expression of MES phenotype marker protein CD44 was lower in the ARPC1B knockdown group than in control group, while a higher protein level of CD44 was detected in ARPC1B overexpression group than in control group (Fig. 3C and Fig. S5A, B). TUNEL staining revealed that sh-ARPC1B group exhibited a higher apoptotic rate than control group after IR treatment, while the ARPC1B overexpression group had a lower apoptosis rate than the control group (Fig. 3D and Fig. S5A, B). H&E staining revealed that tumor invasion was inhibited in the sh-ARPC1B group, whereas opposite result was expected in ov-ARPC1B group versus control group (Fig. S5C). In terms of survival analysis, knockdown of ARPC1B significantly prolonged the overall survival of mice in both radiotherapy and non-radiotherapy cohorts, whereas overexpression of ARPC1B shortened the overall survival of GSC 8-11 xenograft mice in both radiotherapy and non-radiotherapy groups (Fig. 3E). The above results suggested that ARPC1B promoted the progression and radiotherapy resistance of GSCs in vivo.

### ARPC1B maintains the stability of γ-interferon inducible protein 16 (IFI16) and Human antigen R (HuR)

**Fig. 4:**
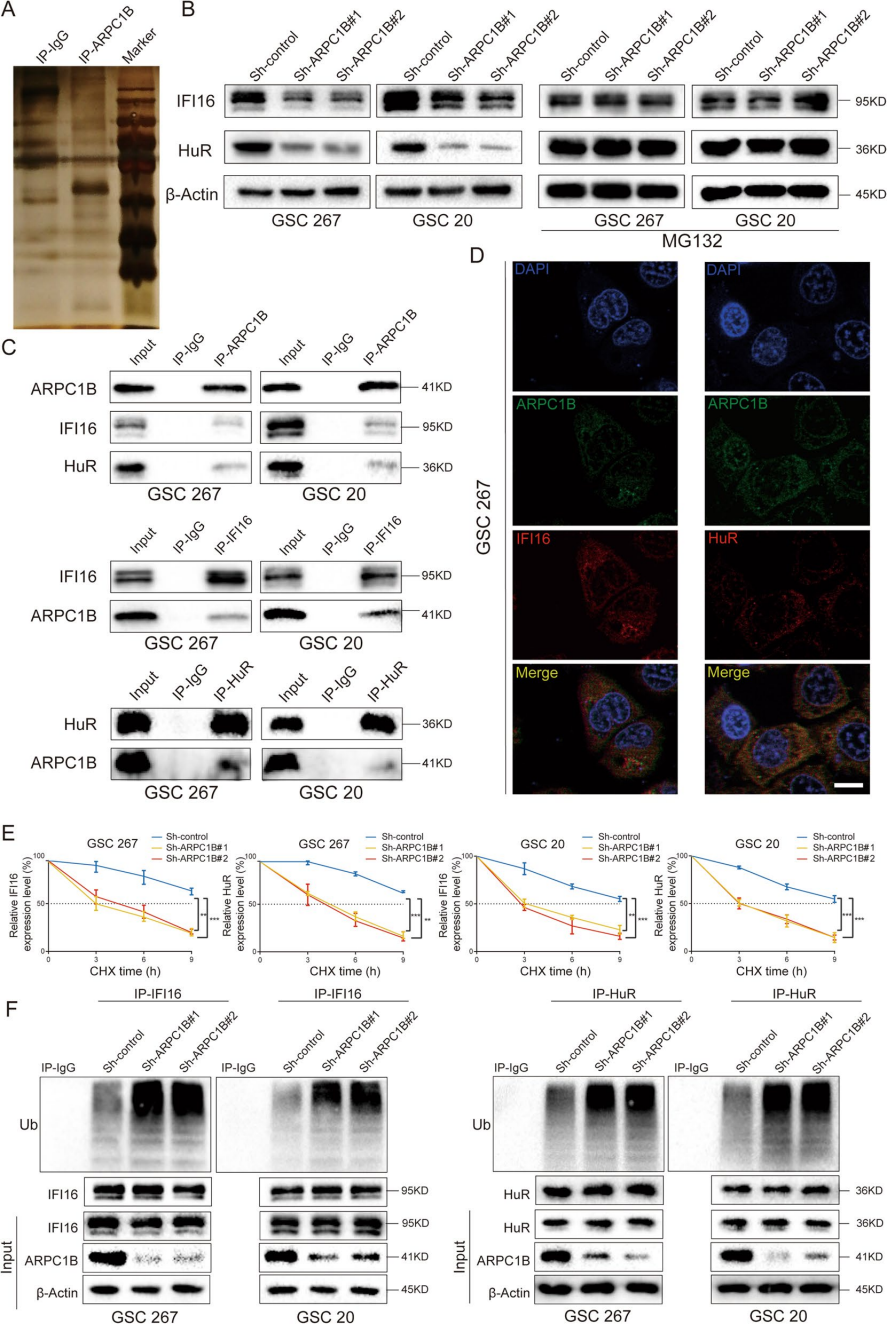
ARPC1B maintains the stability of IFI16 and HuR by direct binding. **A** ARPC1B-interacting proteins in GSC 267 cells were separated by SDS–PAGE for silver staining and mass spectrometry analysis. **B** The protein expression of IFI16 and HuR after ARPC1B knockdown with or without MG132 treatment (10 µM, 12 h). **C** Endogenous reciprocal Co-IP experiment testing the interaction between ARPC1B and IFI16 or HuR in GSC 267 and GSC 20 cells. **D** Co-IF staining showing the distribution of ARPC1B with IFI16 or HuR in GSC 267 cells. Scale bar, 5 μm. **E** The effect of ARPC1B knockdown on IFI16 and HuR protein levels in GSC 20 and GSC 267 cells treated with 100 μg/ml CHX for indicated times. **F** The ubiquitylation of IFI16 and HuR in GSC 20 and GSC 267 cells upon knockdown of ARPC1B. GSCs were pretreated with MG132 (10 µM) for 6 h before cell lysates were collected

To further investigate the mechanism of ARPC1B on GSC phenotype and IR resistance, an IP assay was performed, and proteins interacting with ARPC1B were detected by mass spectrometry analysis (Fig. 4A). A total of 74 proteins were examined at high levels in ARPC1B antibody-enriched samples and at extremely low levels in IgG control samples (Table S2). Given that ARPC1B is mainly distributed in the cytoplasm, we excluded some intranuclear proteins. We then ranked the remaining proteins according to their abundance in the IP-ARPC1B group and reviewed a large number of references. A total of 6 proteins interacting with ARPC1B were selected for the follow-up studies.

After knockdown of ARPC1B in GSC 20 and GSC 267 cells and overexpression of ARPC1B in GSC 8-11 cells, we found that IFI16 and HuR remained consistent with changes in ARPC1B, while the other four proteins did not show significant changes (Fig. 4B, Fig. S5D and Fig. S6B). To further validate the effect of ARPC1B on IFI16 and HuR, we performed IHC on tissue sections from mice with different interventions. The protein levels of IFI16 and HuR were lower in the ARPC1B knockdown group than in control group, while a higher protein level of IFI16 and HuR were detected in ARPC1B overexpression group than in control group (Fig. S6E). Then, we demonstrated again that IFI16 and HuR interacted with ARPC1B by a co-IP assay (Fig. 4C). In addition, co-IF staining demonstrated that ARPC1B colocalized with IFI16 and HuR in cytoplasm (Fig. 4D and Fig. S6A). Finally, IFI16 and HuR, which bind to and are regulated by ARPC1B, were obtained by stepwise screening.

IFI16 is an important double-stranded DNA (dsDNA) sensor that plays an essential role in innate immune response against viral infection [46]. The DNA-binding protein IFI16 can activate STING in a noncanonical manner a (cGAS-independent manner), further leading to activation of NF-κB signaling [47]. HuR, also known as ELAV1, is an RNA-binding protein. HuR-regulated genes are involved in apoptosis, tumorigenesis, radiotherapy resistance, and hypoxia [48]. In addition, HuR counteracts the translational repression of STAT3 by binding mRNA [49].

We performed qRT–PCR and observed that knockdown of ARPC1B in GSC 20 and GSC 267 cells or overexpression of ARPC1B in GSC 8-11 cells had no statistically significant impact on mRNA expression of either IFI16 or HuR (Fig. S6C). Because treatment with MG132, a proteasome inhibitor, could restored the protein levels of IFI16 and HuR upon knockdown of ARPC1B in GSCs (Fig. 4B), we hypothesized that ARPC1B exerted mechanistic effects by affecting protein ubiquitination. Then, CHX experiments showed that knockdown of ARPC1B significantly affected stabilization of IFI16 and HuR in GSC 20 as well as GSC 267 cells (Fig. 4E and Fig. S6D). Furthermore, a co-IP experiment demonstrated that the ubiquitylation levels of IFI16 and HuR were markedly elevated through ARPC1B knockdown (Fig. 4F). Collectively, our results demonstrated that ARPC1B regulated the expression of IFI16 and HuR by affecting protein ubiquitination modification.

### ARPC1B interacted with Pyrin of IFI16 and RRM2 of HuR

**Fig. 5:**
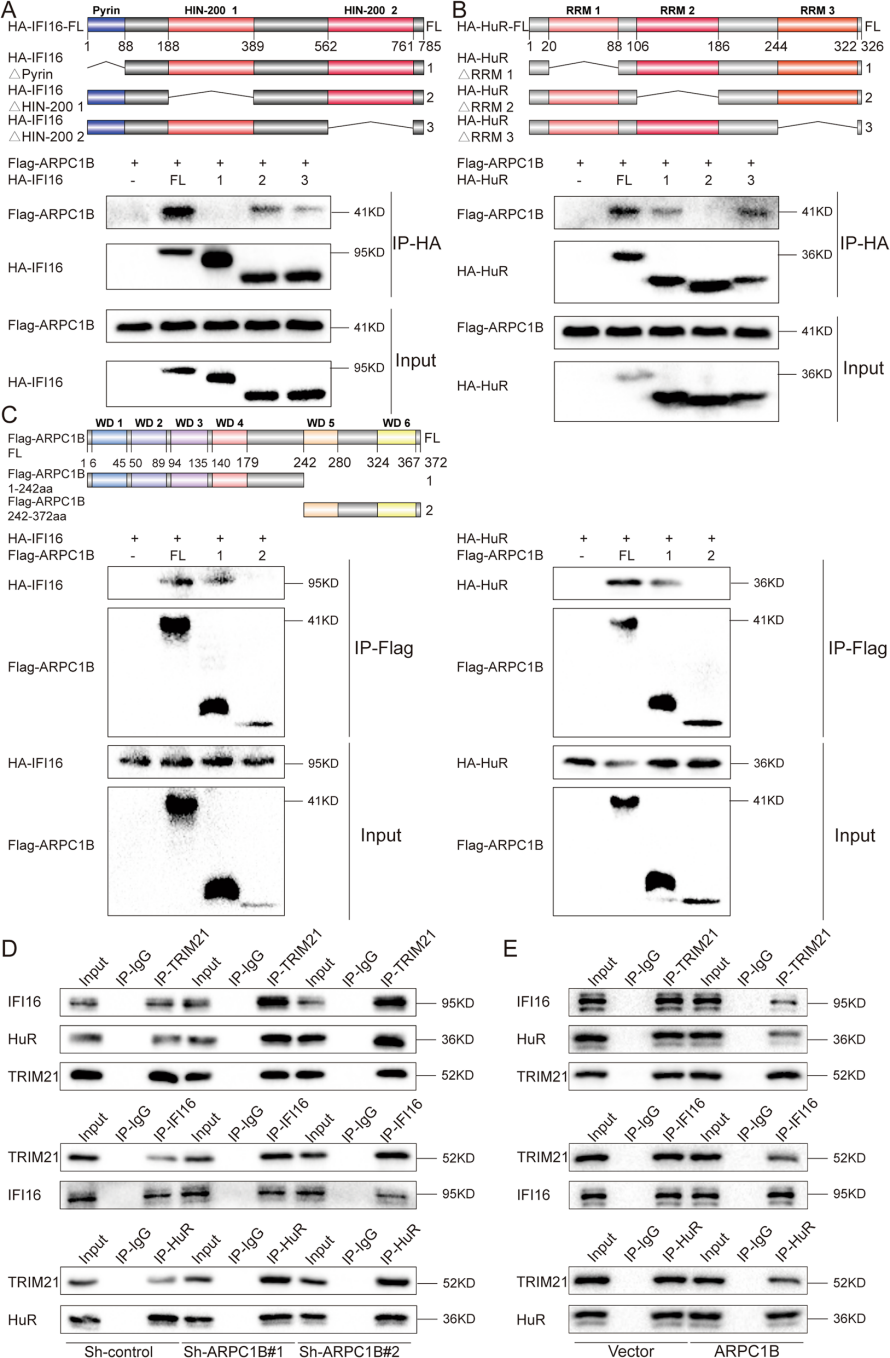
ARPC1B disrupted the TRIM21-mediated IFI16 and HuR ubiquitylation and degradation. **A**, **B** Co-IP assays were performed with an anti-HA antibody on cell lysates from HEK293 cells transfected with Flag-ARPC1B alone or together with the indicated HA-IFI16 (**A**) or HA-HuR (**B**) constructs. The upper panel is a schematic representation of wild-type sequences and the indicated deletion mutants. **C** Western blot analysis of co-IPs in lysates of HEK293 cells transfected with the indicated Flag-ARPC1B constructs together with HA-IFI16 (left panel) or HA-HuR (right panel). The upper panel is a schematic representation of wild-type ARPC1B and the two indicated mutants. **D**, **E** Co-IP analysis revealing the impact of ARPC1B knockdown (**D**) or ARPC1B overexpression (**E**) on the interaction between TRIM21 and IFI16 or TRIM21 and HuR

Protein domains (PDs) are regions of proteins with specific spatial structures and independent functions, which are the key functional units for proteins to perform their biological functions. In a given protein, identification of the structural domains presented and the detailed functions of the domains provides insight into the function of the protein [50]. To determine the potential structural domains of IFI16 and HuR that interact with ARPC1B, we designed a series of deletion mutant constructs and performed co-IP assays. For the interaction of IFI16 with ARPC1B, the Pyrin domain in IFI16 interacted specifically with ARPC1B (Fig. 5A). For the analysis of HuR, the RPM2 region in HuR was responsible for the interaction with ARPC1B (Fig. 5B). Additionally, two fragments of ARPC1B were designed due to the repetitive structural domain of ARPC1B (WD1-6), and amino acid regions 1–242 in ARPC1B interacted with IFI16 and HuR, respectively (Fig. 5C). Thus, we identified the detailed regions responsible for the interaction of ARPC1B with IFI16 and HuR, which could provide guidance for the future development of drugs to interfere with molecular interactions. The protein sequences were showed by Illustrator for Biological Sequences (IBS, http://ibs.biocuckoo.org/download.php). Collectively, we identified Pyrin and RRM2 as two key domains involved in ARPC1B-IFI16/HuR interactions, which were implicated in the PMT and radiotherapy resistance for GSCs. Our results may ensure more effective precision oncology and provide insights into the development of targeted drugs.

### ARPC1B disrupted the tripartite motif containing 21 (TRIM21)-mediated IFI16 and HuR ubiquitylation and degradation

The ubiquitin–proteasome system (UPS) is one of most prevalent types of posttranslational modifications and plays an essential role in various disorders, including cancer [51]. Although we discovered that ARPC1B regulated the stability of IFI16 and HuR by affecting posttranslational ubiquitination modifications, the specific ubiquitinating enzymes involved in this process are still unclear. To explore the key molecules that mediate the degradation of IFI16 and HuR, we identified a potential E3 ubiquitin ligase, TRIM21 [46, 52, 53]. TRIM21, as a member of the RING-finger ubiquitin E3 ligase family, has been reported to induce ubiquitinated degradation of either IFI16 or HuR [46, 53]. Intriguingly, Song et al. demonstrated that TRIM21 interacted with the PYD domain of IFI16, and Guha et al. revealed that the PRM2 domain of HuR was implicated in TRIM21-induced ubiquitination degradation. Combining these previous studies and our available results, we hypothesized that ARPC1B competed with TRIM21 for binding to IFI16 and HuR, thereby counteracting TRIM21-mediated degradation. First, we found that the effects of ARPC1B knockdown on IFI16 and HuR could be rescued by TRIM21 knockdown in GSC 20 and GSC 267 cells (Fig. S7A). In addition, knockdown of TRIM21 in GSC 267 was found to significantly reverse the effect of ARPC1B inhibition on IFI16 and HuR ubiquitination (Fig. S7D). Finally, a series of co-IP essays were conducted to verify the influence of ARPC1B on TRIM21/IFI16 or TRIM21/HuR interactions. The results demonstrated that knockdown of ARPC1B apparently promoted TRIM21/IFI16 as well as TRIM21/HuR interactions (Fig. 5D), whereas overexpression of ARPC1B in GSC 8-11 disrupted TRIM21/IFI16 and TRIM21/HuR interactions (Fig. 5E).

### Overexpression of IFI16 or HuR reactivated the NF-κB and STAT3 signaling pathways in sh-ARPC1B GSCs

The activation of STAT3 and NF-κB signaling pathways driving PMT and radiotherapy resistance has been extensively studied and elucidated. Our fundings demonstrated that knockdown of ARPC1B in GSC 20 and GSC 267 cells reduced protein levels of p-STAT3, STAT3 and p-P65, whereas overexpression of ARPC1B in GSC 8-11 cells elevated protein levels of p-STAT3, STAT3 and p-P65 (Fig. S7B).

**Fig. 6:**
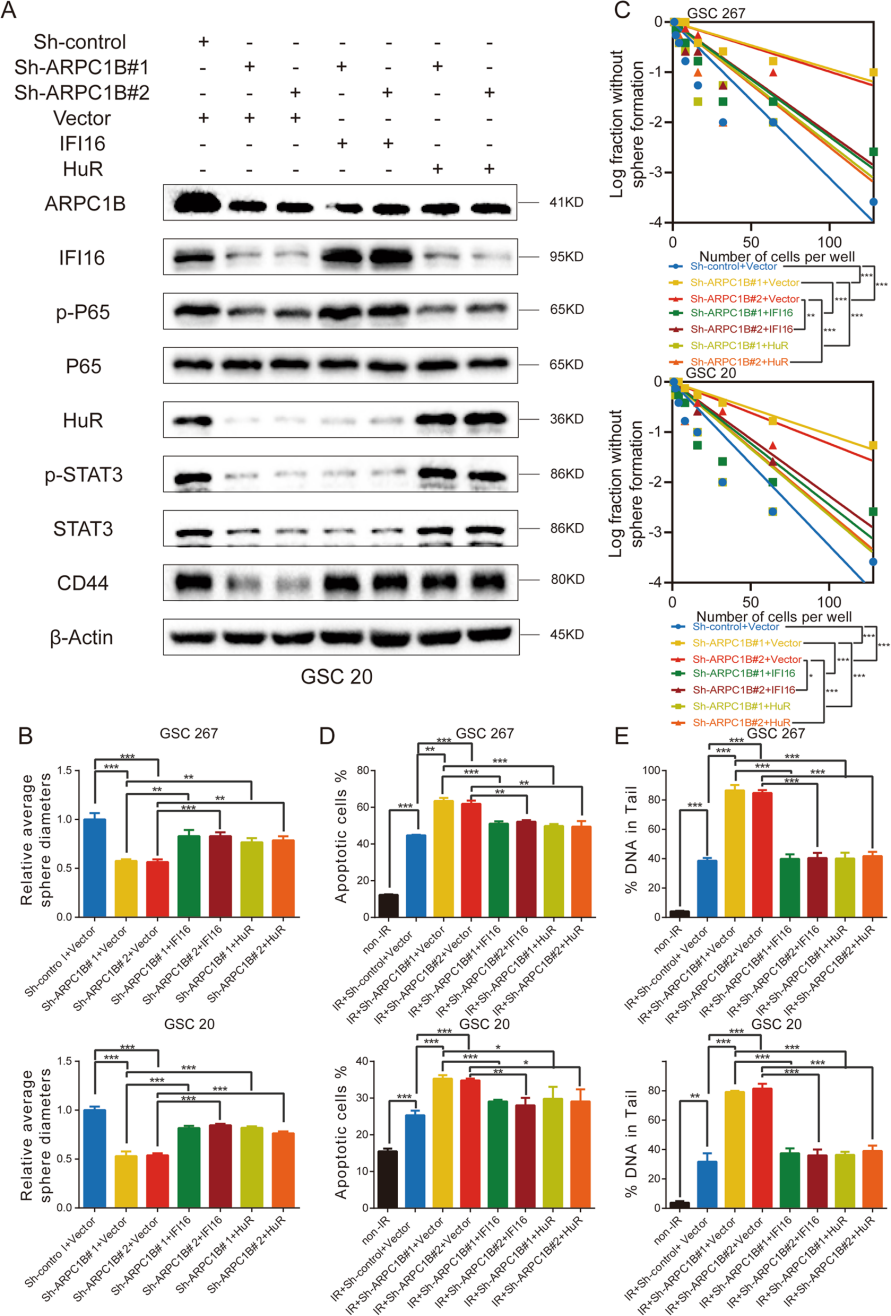
Overexpression of IFI16 or HuR reactivated the STAT3 and NF-κB signaling pathways in sh-ARPC1B GSCs. **A** Western blotting analysis of protein expression of ARPC1B, IFI16, HuR, P65, p-P65, STAT3, p-STAT3 and CD44 in GSC 20 cells treated with the indicated interventions. **B** The quantification of tumor sphere formation of GSC 267 and GSC 20 cells treated with the indicated interventions. **C** Limiting dilution assays of GSC 267 and GSC 20 cells treated with the indicated interventions. **D** Quantification of the apoptosis rates of GSC 267 and GSC 20 cells treated with the indicated interventions. **E** Quantification of DNA damage evaluated by the comet assay in GSC 267 and GSC 20 cells treated with the indicated interventions

We then performed a series of experiments to determine whether ARPC1B functions through IFI16 and HuR. In sh-ARPC1B GSC 20 and GSC 267 cells, overexpression of IFI16 restored the protein levels of p-P65, whereas overexpression of HuR restored the protein expression of STAT3 and p-STAT3. In addition, the expression of CD44 was also rescued by IFI16 and HuR overexpression (Fig. 6A and Fig. S7C). For the tumor sphere formation assay and limiting dilution assay, overexpression of IFI16 and HuR both restored the self-renewal and tumorigenesis ability of sh-ARPC1B GSCs, respectively (Fig. 6B, C and Fig. S8A).

Then, apoptosis and comet experiments were conducted to evaluate the resistance of GSCs to radiotherapy among different groups. The apoptosis rate was further increased by knockdown of ARPC1B, while overexpression of IFI16 or HuR counteracted the adverse effect of ARPC1B knockdown in GSC 20 and GSC 267 cells (Fig. 6D and Fig. S8B). Additionally, the comet assay showed that knockdown of ARPC1B significantly increased DNA damage, while overexpression of IFI16 or HuR counteracted the effect of ARPC1B knockdown on DNA damage in GSC 20 and GSC 267 cells (Fig. 6E and Fig. S8C). Collectively, our results demonstrated that ARPC1B promoted PMT and radiotherapy resistance by affecting IFI16 and HuR.

### ARPC1B expression is reflective of sensitivity to the ATR inhibitor AZD6738

**Fig. 7:**
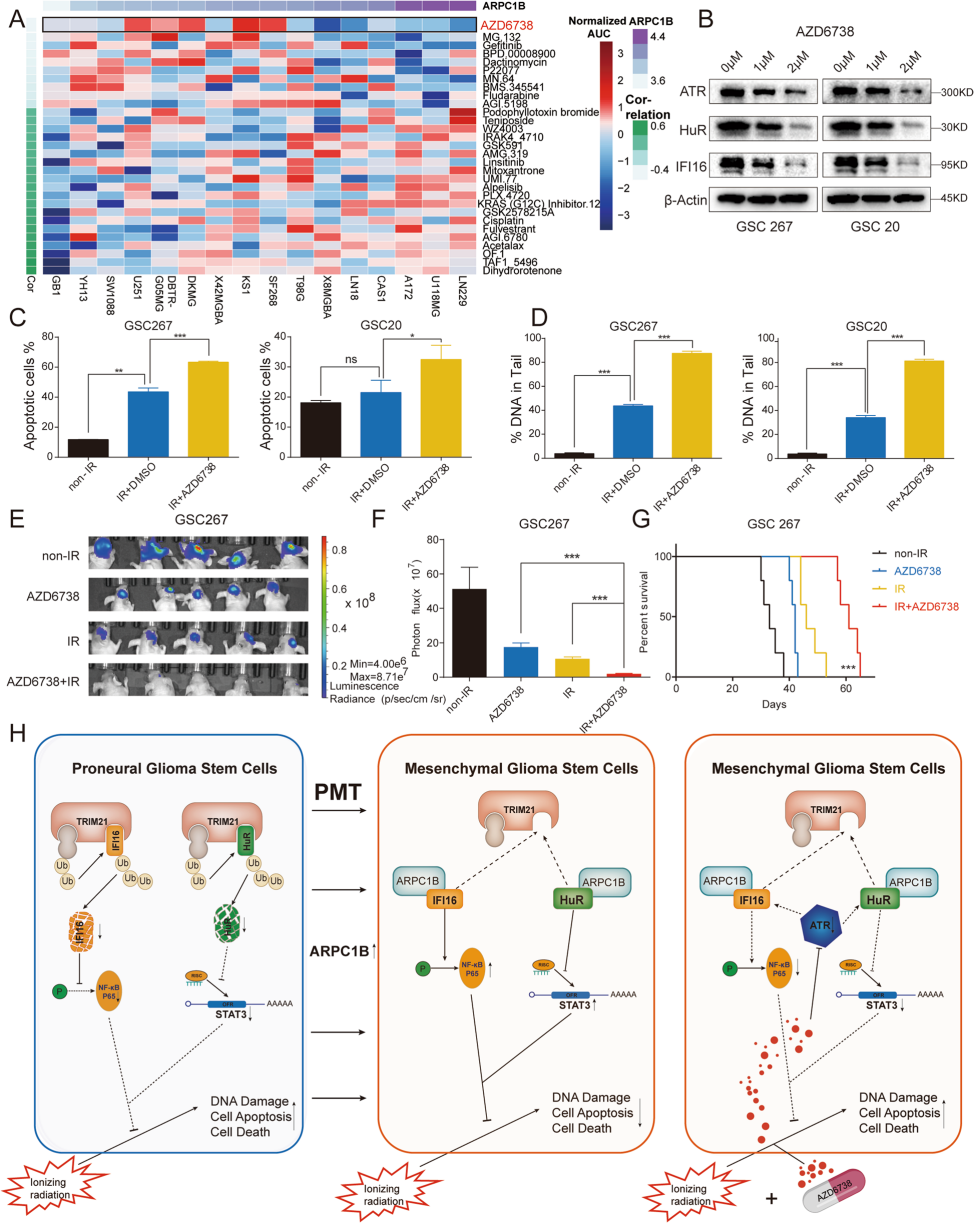
ARPC1B expression is reflective of sensitivity to the ATR inhibitor AZD6738. **A** Heatmap visualizing the difference in sensitivity of cell lines with high and low expression of ARPC1B to the indicated drugs. **B** Western blot analysis of ATR, IFI16 and HuR protein expression upon AZD6738 treatment in GSCs. β-Actin served as the control. **C** Quantification of apoptosis rates of GSC 267 and GSC 20 cells with the indicated treatments. **D** The DNA damage assessed by the comet assay was quantified for GSC 267 and GSC 20 cells treated with the indicated interventions. **E** Bioluminescence imaging of tumor size on day 30 in GSC 267 xenograft nude mice treated with the indicated interventions. **F** The quantification of photon counts on day 30 of the GSC 267 xenografts. **G** Kaplan–Meier curves visualizing the survival of GSC 267 xenograft mice in different treatment groups. **H** The graphical model of this study. ARPC1B was upregulated in MES-GSCs and overexpression of ARPC1B in PN-GSCs facilitated the transition to MES-GSCs. ARPC1B suppressed TRIM21-mediated degradation of IFI16 and HuR, thereby activating NF-κB and STAT3, which promoted PMT and counteracted the tumoricidal effect of IR. AZD6738, as a selective ATR inhibitor, exhibited promising anti-GSCs effect in combination with IR

Since ARPC1B is highly variably expressed in different phenotypes of GBM cells, we examined the relationship between ARPC1B expression and sensitivity to therapeutic agents. Interestingly, the GBM cell lines expressing higher levels of ARPC1B were strikingly more sensitive to AZD6738 (synonyms: ceralasertib) (Fig. 7A, Fig. S9A and Table S3), which is a selective inhibitor of ATR [54]. A growing amount of research demonstrated that AZD6738 exhibits antitumor activity as a monotherapy or in combination with other agents or radiation therapy [36, 38, 39, 55–57]. In addition, the activation of ATR has been reported to be implicated in the upregulation of IFI16 and HuR [58, 59]. Our results also demonstrated that the protein levels of ATR, IFI16 and HuR decreased significantly with increasing concentrations of AZD6738 treatment (Fig. 7B). CCK-8 cell viability assay was conducted to identify the IC_50_ of AZD6738 in GSC 20 (IC_50_ = 1.531 μM) as well as GSC 267 cells (IC_50_ = 1.609 μM) (Fig. S9B). In vitro experiments found that GSCs treated with AZD6738 exhibited higher sensitivity to radiotherapy. The number of apoptotic cells and DNA damage were significantly increased in the AZD6738 combined with radiotherapy group (Fig. 7C, D and Fig. S9C). For GSC 8-11, while overexpression of ARPC1B counteracted the adverse effects caused by IR intervention, the administration of AZD6738 further enhanced the rate of apoptosis and DNA damage (Fig. S9D). In vivo experiments further found that the combination of AZD6738 with radiotherapy apparently reduced tumor size and prolonged overall survival of mice (Fig. 7E-G and Fig. S9F, G). TUNEL staining in sections from the GSC 267 xenografts revealed a higher percentage of apoptotic cells in the combination therapy group (Fig. S9E). Finally, a graphical model visualized the main aspects of this study (Fig. 7H).

## Discussion

A deeper perspective on the various sources and modulation of heterogeneity is essential to treat and overcome GBM recurrence. In addition to the divergence of transcriptional phenotypes reflected in intra- or inter-tumor heterogeneity, the existence of GSCs and GBM cells at different stages of differentiation illustrates the heterogeneity of developmental stages [57]. That is, MES-GSCs and PN-GSCs, as well as their more differentiated progeny, coexist in GBMs and comprise hybrid hierarchies.

Additionally, heterogeneity is mainly maintained by cellular plasticity, while genetic events that drive the properties of the most common cell stages appear to incompletely explain cell phenotype transitions in GBM. More comprehensive mechanisms, including alterations of intracellular pathways and extracellular environments resulting from therapeutic intervention or tumor progression, drive plasticity and a skew toward specific cellular stages.

Because the PN-to-MES transition is considered a marker of recurrent GBM and resistance to multiple therapies, the mechanisms mediating PMT have attracted much attention. For example, Mutsuko et al. demonstrated that upon exposure to IR, GSCs enriched in the PN gene signature transformed into GSCs enriched in the MES gene signature through C/EBPβ [8]. Ashwin et al. revealed that both silencing ASCL1 expression and overexpressing NDRG1 expression in PN-GSCs could promote PMT [13]. The TME also plays an important role in PMT [60]. Toshiro et al. found that the MES state of GBM was implicated in macrophage-derived OSM interacting with OSMR/LIFR-GP130 [18]. Our previous study also indicated that macrophage-derived sEVs could act as critical modulators of PMT in GSCs. Above all, numerous studies and possible mechanisms suggest that a comprehensive combination of factors contributes to PMT, which remains to be explored step by step.

In this study, we analyzed the role of ARPs in GBM phenotypes and prognosis, and identified that ARPC1B was markedly upregulated in the MES phenotype and predicted a poor outcome. Both loss-of- and gain-of-function assays in GSCs demonstrated that APRC1B promoted PMT and radiotherapy resistance. In detail, ARPC1B interacted with IFI16 and HuR, blocking TRIM21-mediated ubiquitinated degradation of IFI16 and HuR. ARPC1B promoted PMT and radiotherapy resistance through IFI16 and HuR, which are responsible for activating the NF-κB and STAT3 signaling pathways, respectively. Moreover, assessment of ARPC1B expression could reflect the sensitivity to the ATR inhibitor AZD6738, which has shown promising antitumor effects in combination with radiotherapy.

Previously, it was shown that ARPC1B played an important role in macrophage-tumor intertwining, whereas our results demonstrated that ARPC1B was critical for the activation of intrinsic key pathways of GSCs, which is an important complement to ARPC1B for PMT. We found that ARPC1B promoted PMT and radiotherapy resistance by maintaining the stability of IFI16 and HuR, which were implicated in activation of NF-κB and STAT3 pathways, respectively. IFI16 integrates tightly with the cGAS-cGAMP-STING signaling pathway and synergizes with cGAMP to promote STING activation, thereby shaping the local immune response to exogenous DNA and DNA viruses [61].

Additionally, intrinsic nuclear DNA damage also causes activation of STING and innate immune responses, which are independent of the cGAS signaling pathway. IFI16 together with ATM and PARP-1 mediates the noncanonical activation of STING. Meanwhile, activated STING signaling is further implicated in the activation of NF-κB signaling. In addition to the function of IFI16 in the STING/NF-κB axis, Cindy et al. found that activation of the ATR/CHK1 pathway was related to an increased number of cytoplasmic ssDNA and micronuclei, which further activated the IFI16/STING pathway [58]. Their work revealed the upstream ATR of IFI16, which provided insights for our subsequent drug design and analysis.

HuR is reported to counteract miR-330 to promote STAT3 translation [49]. In addition, HuR is responsible for IL-6 mRNA stability, which further activates the JAK1/STAT3 signaling pathway [62]. In terms of the regulation of HuR, Hyeon et al. reported that the function of HuR was regulated by ATM/ATR through Chk1 and Chk2 [59]. It is clear that the upstream regulation of both IFI16 and HuR is implicated in the activation of ATR. Intriguingly, the drug we screened based on the expression profile of ARPC1B, AZD6738, is also coincidentally an inhibitor of ATR.

ATR is implicated in coordinating the DNA damage response (DDR) induced by DNA replication-related stress and cell cycle checkpoints [33]. The activation of ATR is essential for fork stabilization in response to replication stress and adverse stress-induced DNA damage [63]. Therefore, targeting ATR has exhibited promising antitumor effects, and a variety of ATR inhibitors have been developed. AZD6738, an oral inhibitor of ATR, has been studied as a monotherapy or in combination with radiotherapy, chemotherapy or immunotherapy. Our results indicated that AZD6738 in combination with radiotherapy exhibited excellent antitumor activity in vitro and in vivo. As previously discussed, the regulation of IFI16 and HuR is involved in the ATR pathway, whereas AZD6738 has been validated to target ATR-induced activation of IFI16 and HuR. Therefore, AZD6738 has the potential to counteract ARPC1B-induced resistance to radiotherapy in GSCs. We demonstrated that AZD6738 was implicated in the suppression of two downstream molecules of ARPC1B, IFI16 and HuR. That is, in addition to the cellular sensitivity to AZD6738 being reflected by the expression of ARPC1B, AZD6738 also has the potential to inhibit PMT and resistance to radiotherapy by affecting IFI16 and HuR.

## Conclusions

In conclusion, we identified ARPC1B, which is significantly upregulated in MES-GBM/GSCs and is correlated with a poor prognosis. ARPC1B promotes PMT and radiotherapy resistance by inhibiting TRIM21-mediated degradation of IFI16 and HuR, thereby activating the NF-κB and STAT3 signaling pathways, respectively. AZD6738 in combination with radiotherapy exhibited potent anti-GSC effects. Our findings expand the understanding of the heterogeneity and plasticity of GBM and provide a potential therapeutic strategy for GBM treatment.

## Supplementary Information

**Additional file 1: Figure S1.** (A) The expression of ARPs among mesenchymal (MES), proneural (PN), and classical (CL) phenotypes in TCGA GBM. (B) Kaplan–Meier curves visualizing the overall survival of CCGA-GBM patients stratified according to expression of ARPs. (C) The expression of ARPs among MES, PN, and CL phenotypes in CGGA GBM. (D) GSEA exhibited a positive correlation between ARPC1B expression and MES phenotypes, and a negative correlation with PN phenotypes. (E) Single-cell RNA sequencing of GSE138794 visualizing the expression of ARPs other than ARPC1B. **Fig. S2.** (A) Correlation analysis of ARPC1B with CD44, YKL-40, OLIG2 and SOX2 in TCGA-GBM and CGGA-GBM, respectively. (B) Western blot analysis of ARPC1B and SOX2 protein levels in GSC 8-11 overexpressing ARCP1B. (C, D) Representative images and quantification of tumor sphere formation of GSC 8-11 (C) and GSC 11 (D) transduced with vector or ARPC1B. Scale bar, 100μm. (E) The protein expression of ARPC1B in GSC 20 and GSC 267 under different IR dose treatments. **Fig. S3.** (A) Flow cytometric analysis showing the effect of ARPC1B overexpression on the apoptosis in IR-treated (6Gy) GSC 8-11 cells. The right panels showing the quantification of apoptosis rate. (B) Representative images and quantification of comet assay showing the effect of ARPC1B overexpression on DNA damage of GSC 8-11 with IR treatment (6 Gy). Scale bar, 20μm. (C) Representative images and quantification of γ-H2AX IF staining showing the effect of ARPC1B knockdown on DNA damage of GSC 267 and GSC 20 with IR treatment (6 Gy). Scale bar, 40μm. (D) Representative images and quantification of γ-H2AX IF staining in GSC 8-11. Scale bar, 40μm. (E) Cell-cycle analysis of GSC 267, GSC 20 and GSC 8-11 in different treatment groups. The proportions of cells arrested in G2/M phase were quantified (right panel). **Fig. S4.** (A, B) Bioluminescence imaging of tumor size on day 7 in sh-control, sh-ARPC1B#1 and sh-ARPC1B#2 GSC 267 (A) or GSC 20 (B) xenograft nude mice in indicated groups. The right panel shows the quantification of photon counts of GSC 267 and GSC 20 xenografts. (C, D) Bioluminescence imaging of tumor size on day 7 (C) and day 30 (D) in Vector or ARPC1B-transfected GSC 8-11 xenograft nude mice receiving or exempt from IR treatment. The right panel showing the quantification of photon counts of GSC 8-11 xenografts. **Fig. S5.** (A) Representative images and quantification of IHC staining for CD44 in sections of non-IR GSC 267 xenografts (upper, scale bar, 200μm), and TUNEL staining in sections of IR treated GSC 267 xenografts (lower, scale bar, 200μm). (B) Representative images and quantification of IHC staining for CD44 in sections of non-IR GSC 8-11 xenografts (upper, scale bar, 200μm), and TUNEL staining in sections of IR treated GSC 8-11 xenografts (lower, scale bar, 200μm). (C) Representative images of H&E staining in sections from indicated xenografts. Scale bar, 400μm. (D) Western blotting analysis of protein levels of DBN1, ACTN4, FLNA, and CORO1C upon knockdown of ARPC1B in GSC 20 and GSC 267, or overexpression of ARPC1B in GSC 8-11. **Fig. S6.** (A) Co-IF staining exhibiting the distribution of ARPC1B with IFI16 or HuR in GSC 20. Scale bar, 5μm. (B) The protein levels of IFI16 and HuR after overexpression of ARPC1B in GSC 8-11. (C) The mRNA expression of IFI16 and HuR assessed by qRT-PCR assay. (D) The protein levels of IFI16 and HuR in sh-control or sh-ARPC1B-GSCs treated with 100μg/ml CHX for indicated times. (E) Representative images and quantification of IHC staining for ARPC1B, HuR and IFI16 in different groups of GSC 267 xenograft sections (scale bar, 200μm). **Fig. S7.** (A) Western blotting analysis showing the effect of TRIM21 knockdown on the protein levels of IFI16 and HuR in sh-ARPC1B GSCs. (B) Western blotting analysis showing the effect of ARPC1B knockdown in GSC 20 and GSC 267, or ARPC1B overexpression in GSC 8-11 on the protein levels of STAT3, p-STAT3, P65, and p-P65. (C) Western blotting analysis of protein levels of ARPC1B, IFI16, HuR, P65, p-P65, STAT3, p-STAT3 and, CD44 in GSC 20 treated with indicated interventions. (D) Western blotting analysis showing that knockdown of TRIM21 could reverse the effect of ARPC1B inhibition on IFI16 and HuR ubiquitination. GSCs were pretreated with MG132 (10 µM) for 6 hours before cell lysates were collected. **Fig. S8.** (A) Representative images of tumor sphere formation of GSC 267 and GSC 20 treated with indicated interventions. Scale bar, 100μm. (B) Representative images of flow cytometry assays showing apoptosis of GSC 267 and GSC 20 treated with indicated interventions. (C) Representative images of comet assays showing DNA damage of GSC 267 and GSC 20 treated with indicated interventions. Scale bar, 20μm. **Fig. S9.** (A) The correlation between the ARPC1B expression and drug sensitivity assessed by Spearman algorithm. (B) CCK-8 assay in GSC 267 and GSC 20 treated with different concentrations of AZD6738 for 48 h. (C) Representative images and quantification of apoptosis assays (upper panel) and comet assays (lower panel, scale bar, 20μm) for GSC 20 and GSC 267 upon treatment with the indicated interventions. (D) The representative images and quantification of apoptosis assays (upper panel) and comet assays (lower panel, scale bar, 20μm) for GSC 8-11 upon treatment with the indicated interventions. The right panels are the quantification of apoptosis rate and DNA damage, respectively. (E) Representative images and quantification of TUNEL staining in sections of GSC 267 xenografts for different groups. Scale bar, 200μm. (F) Bioluminescence imaging of tumor size on day 7 in GSC 267 xenograft nude mice treated with the indicated interventions. (G) The quantification of photon counts on day 7 of the GSC 267 xenografts. **Supplementary Materials and Methods**.

**Additional file 2: Table S1.** Correlation analysis of ARPC1B with PN and MES marker genes (Correlation). **Table S2.** Mass spectrometry result of proteins related with ARPC1B. **Table S3.** The correlation between the ARPC1B expression and drug sensitivity assessed by Spearman algorithm (correlation).

## Data Availability

Publicly available datasets were applied in this research. These resources could be found here: TCGA: https://portal.gdc.cancer.gov/; CGGA: http://www.cgga.org.cn/; GEO: https://www.ncbi.nlm.nih.gov/gds/ (Accession number is GSE138794). Other data obtained and/or analyzed during the current study were available from the corresponding authors on reasonable request.

## Abbreviations

ARP2/3: Actin-related protein-2/3
ARPC1B: Actin related protein 2/3 complex, subunit 1B
ATR: Ataxia telangiectasia mutated and rad3 related
CGGA: Chinese Glioma Genome Atlas
CL: Classical
DDR: DNA damage response
EMT: Epithelial-mesenchymal transition
GBM: Glioblastoma
GSCs: Glioma stem cells
IDH: Isocitrate dehydrogenase
IF: Immunofluorescence
IHC: Immunohistochemical
IFI16: γ-Interferon-inducible protein-16
IR: Ionizing radiation
MES: Mesenchymal
MRs: Master regulators
NPC: Neural progenitor cells
PMT: PN-to-MES transition
PN: Proneural
TCGA: The Cancer Genome Atlas
TME: Tumor microenvironment
