# Supplementary material for: ARPC1B promotes mesenchymal phenotype maintenance and radiotherapy resistance by blocking TRIM21-mediated degradation of IFI16 and HuR in glioma stem cells
Source: J Exp Clin Cancer Res. 2022 Nov 16;41:323. doi: 10.1186/s13046-022-02526-8 (PMC9667586; doi:10.1186/s13046-022-02526-8)
Supplement: Supplementary file 1 — Additional file 1: Figure S1. (A) The expression of ARPs among mesenchymal (MES), proneural (PN), and classical (CL) phenotypes in TCGA GBM. (B) Kaplan–Meier curves visualizing the overall survival of CCGA-GBM patients stratified according to expression of ARPs. (C) The expression of ARPs among MES, PN, and CL phenotypes in CGGA GBM. (D) GSEA exhibited a positive correlation between ARPC1B expression and MES phenotypes, and a negative correlation with PN phenotypes. (E) Single-cell RNA sequencing of GSE138794 visualizing the expression of ARPs other than ARPC1B. Fig. S2. (A) Correlation analysis of ARPC1B with CD44, YKL-40, OLIG2 and SOX2 in TCGA-GBM and CGGA-GBM, respectively. (B) Western blot analysis of ARPC1B and SOX2 protein levels in GSC 8-11 overexpressing ARCP1B. (C, D) Representative images and quantification of tumor sphere formation of GSC 8-11 (C) and GSC 11 (D) transduced with vector or ARPC1B. Scale bar, 100μm. (E) The protein expression of ARPC1B in GSC 20 and GSC 267 under different IR dose treatments. Fig. S3. (A) Flow cytometric analysis showing the effect of ARPC1B overexpression on the apoptosis in IR-treated (6Gy) GSC 8-11 cells. The right panels showing the quantification of apoptosis rate. (B) Representative images and quantification of comet assay showing the effect of ARPC1B overexpression on DNA damage of GSC 8-11 with IR treatment (6 Gy). Scale bar, 20μm. (C) Representative images and quantification of γ-H2AX IF staining showing the effect of ARPC1B knockdown on DNA damage of GSC 267 and GSC 20 with IR treatment (6 Gy). Scale bar, 40μm. (D) Representative images and quantification of γ-H2AX IF staining in GSC 8-11. Scale bar, 40μm. (E) Cell-cycle analysis of GSC 267, GSC 20 and GSC 8-11 in different treatment groups. The proportions of cells arrested in G2/M phase were quantified (right panel). Fig. S4. (A, B) Bioluminescence imaging of tumor size on day 7 in sh-control, sh-ARPC1B#1 and sh-ARPC1B#2 GSC 267 (A) or GSC 20 (B) xenograft nude [file 13046_2022_2526_MOESM1_ESM.docx]

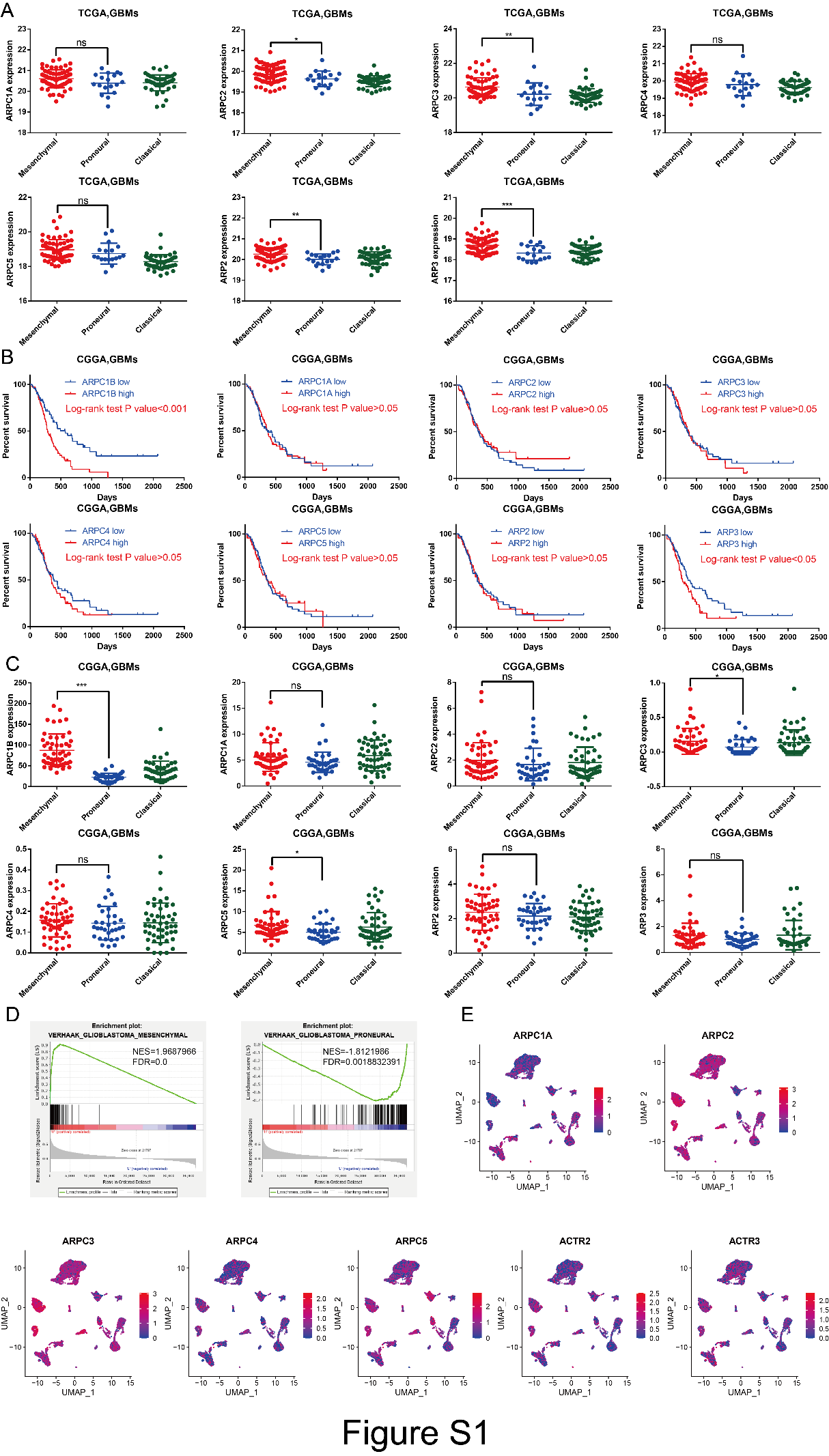


**Figure S1 (A)** The expression of ARPs among mesenchymal (MES), proneural (PN), and classical (CL) phenotypes in TCGA GBM. **(B)** Kaplan–Meier curves visualizing the overall survival of CCGA-GBM patients stratified according to expression of ARPs. **(C)** The expression of ARPs among MES, PN, and CL phenotypes in CGGA GBM. **(D)** GSEA exhibited a positive correlation between ARPC1B expression and MES phenotypes, and a negative correlation with PN phenotypes. **(E)** Single-cell RNA sequencing of GSE138794 visualizing the expression of ARPs other than ARPC1B.


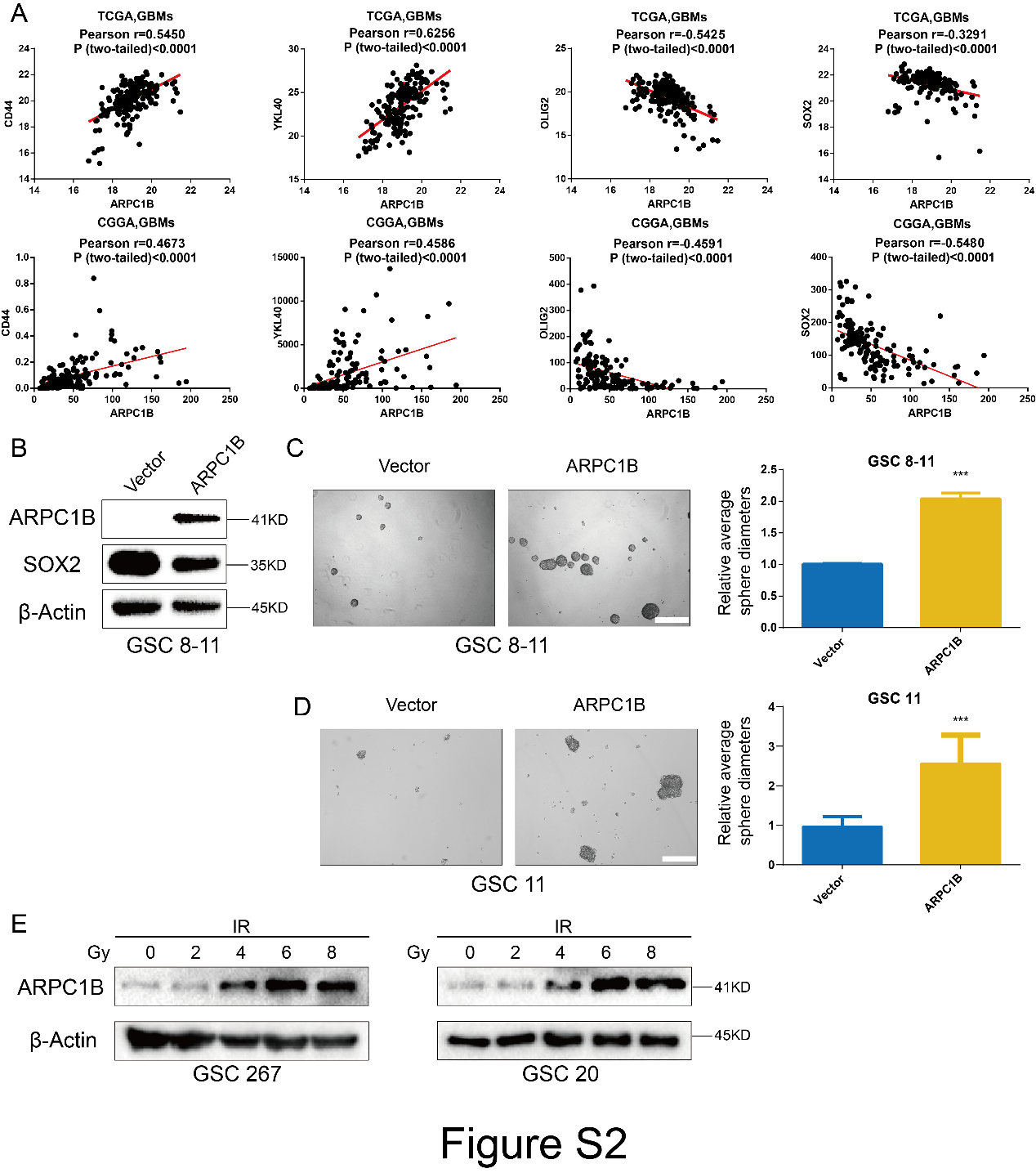


**Fig. S2 (A)** Correlation analysis of ARPC1B with CD44, YKL-40, OLIG2 and SOX2 in TCGA-GBM and CGGA-GBM, respectively. **(B)** Western blot analysis of ARPC1B and SOX2 protein levels in GSC 8-11 overexpressing ARCP1B. **(C, D)** Representative images and quantification of tumor sphere formation of GSC 8-11 **(C)** and GSC 11 **(D)** transduced with vector or ARPC1B. Scale bar, 100μm. **(E)** The protein expression of ARPC1B in GSC 20 and GSC 267 under different IR dose treatments.


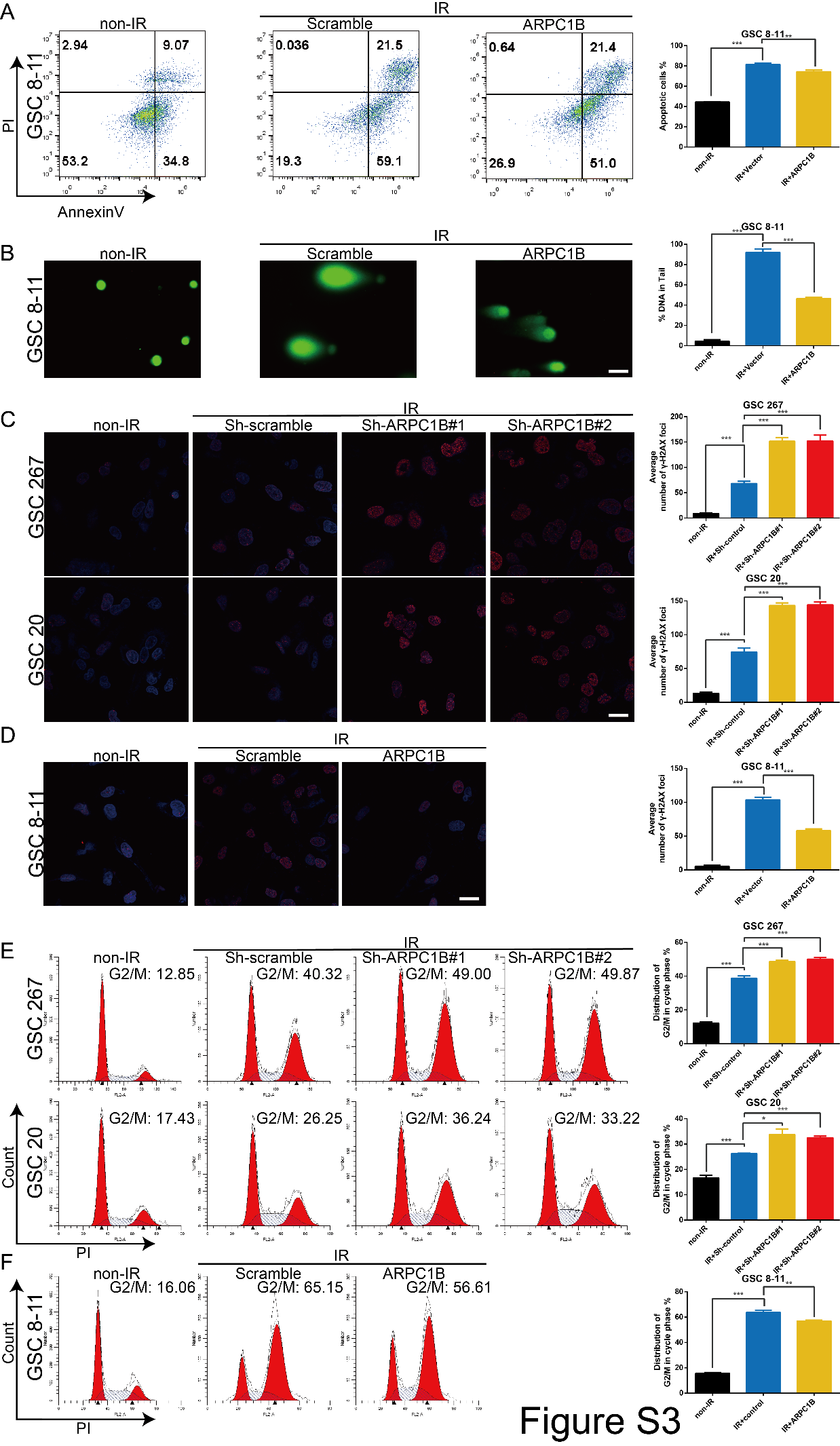


**Fig. S3 (A)** Flow cytometric analysis showing the effect of ARPC1B overexpression on the apoptosis in IR-treated (6Gy) GSC 8-11 cells. The right panels showing the quantification of apoptosis rate. (**B)** Representative images and quantification of comet assay showing the effect of ARPC1B overexpression on DNA damage of GSC 8-11 with IR treatment (6 Gy). Scale bar, 20μm. **(C)** Representative images and quantification of γ-H2AX IF staining showing the effect of ARPC1B knockdown on DNA damage of GSC 267 and GSC 20 with IR treatment (6 Gy). Scale bar, 40μm. **(D)** Representative images and quantification of γ-H2AX IF staining in GSC 8-11. Scale bar, 40μm. **(E)** Cell-cycle analysis of GSC 267, GSC 20 and GSC 8-11 in different treatment groups. The proportions of cells arrested in G2–M phase were quantified (right panel).


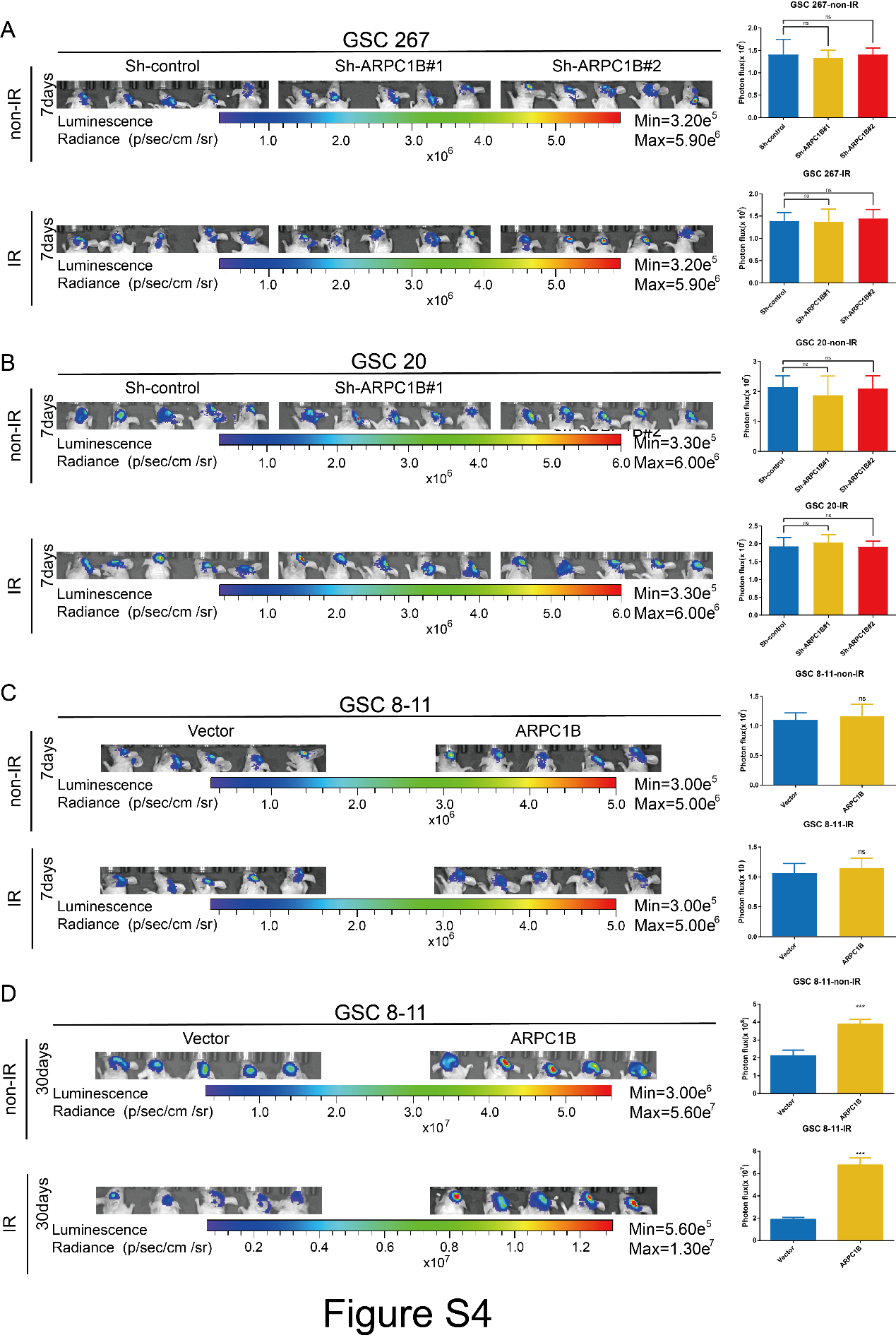


**Fig. S4 (A, B)** Bioluminescence imaging of tumor size on day 7 in shNC, shARPC1B#1 and shARPC1B#2 GSC 267 **(A)** or GSC 20 **(B)** xenograft nude mice in indicated groups. The right panel shows the quantification of photon counts of GSC 267 and GSC 20 xenografts. **(C, D)** Bioluminescence imaging of tumor size on day 7 **(C)** and day 30 **(D)** in Vector or ARPC1B-transfected GSC 8-11 xenograft nude mice receiving or exempt from IR treatment. The right panel showing the quantification of photon counts of GSC 8-11 xenografts.


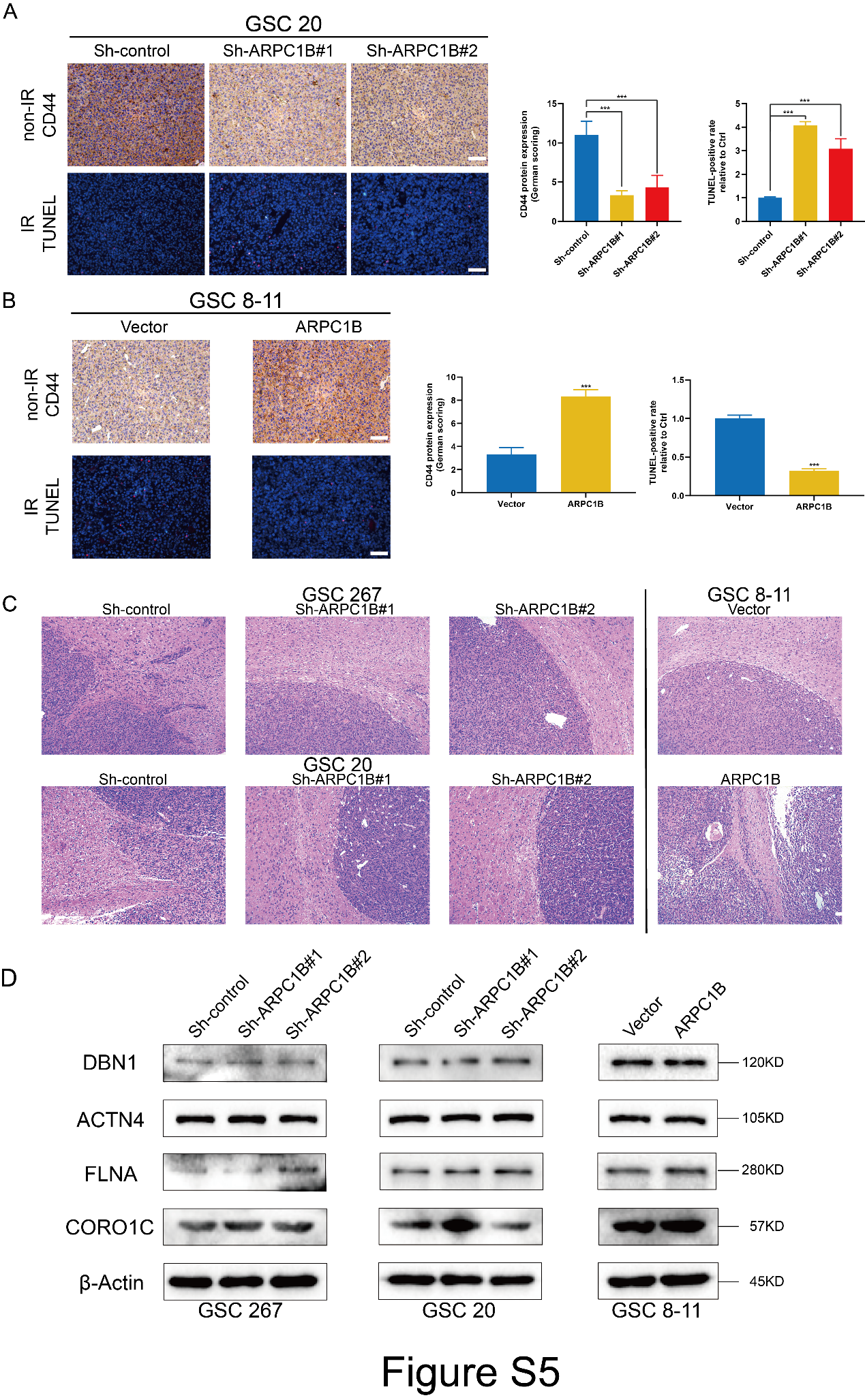


**Fig. S5 (A)** Representative images and quantification of IHC staining for CD44 in sections of non-IR GSC 267 xenografts (upper, scale bar, 200μm), and TUNEL staining in sections of IR treated GSC 267 xenografts (lower, scale bar, 200μm). **(B)** Representative images and quantification of IHC staining for CD44 in sections of non-IR GSC 8-11 xenografts (upper, scale bar, 200μm), and TUNEL staining in sections of IR treated GSC 8-11 xenografts (lower, scale bar, 200μm). **(C)** Representative images of H&E staining in sections from indicated xenografts. Scale bar, 400μm. **(D)** Western blotting analysis of protein levels of DBN1, ACTN4, FLNA, and CORO1C upon knockdown of ARPC1B in GSC 20 and GSC 267, or overexpression of ARPC1B in GSC 8-11.


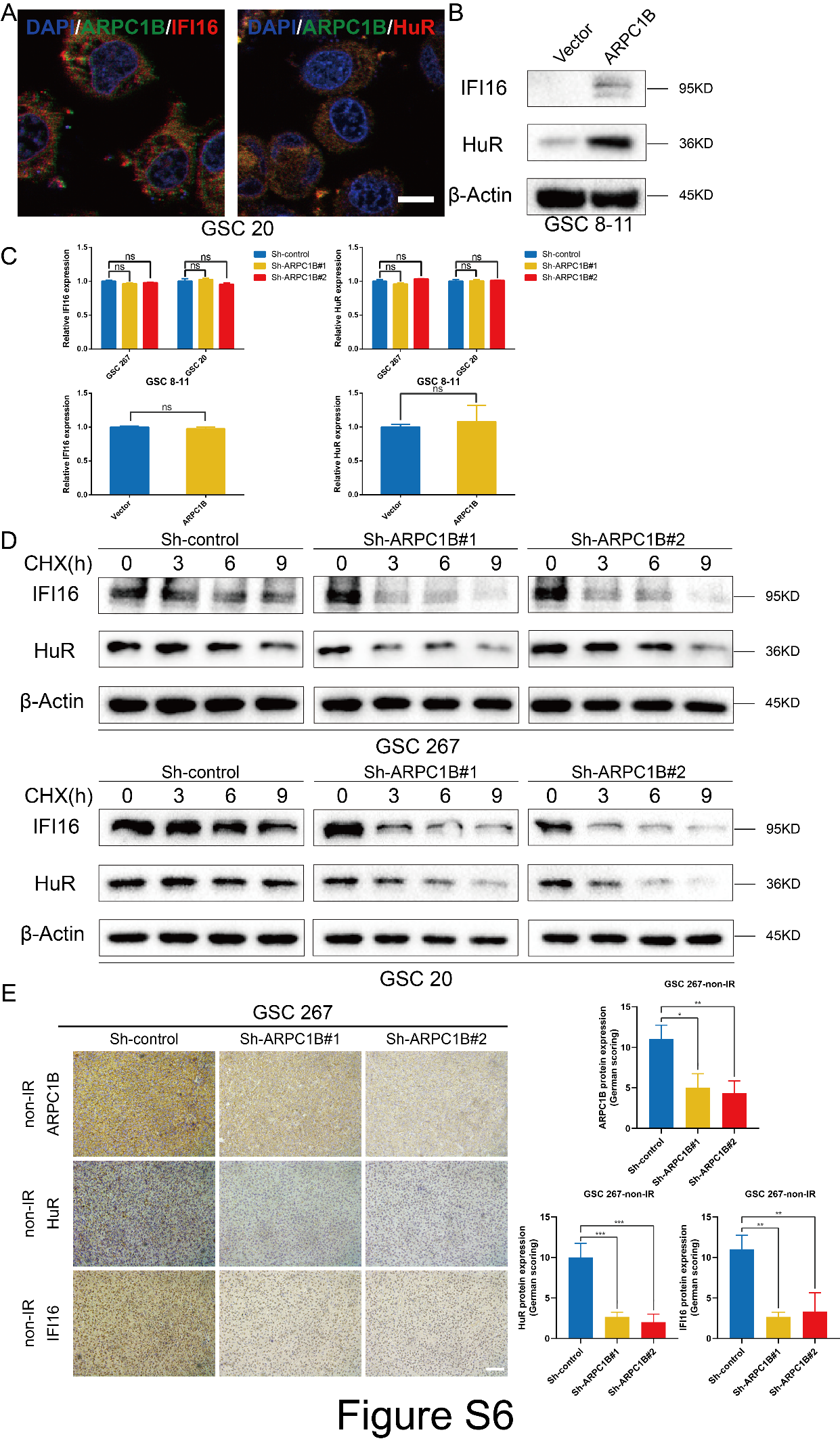


**Fig. S6 (A)** Co-IF staining exhibiting the distribution of ARPC1B with IFI16 or HuR in GSC 20. Scale bar, 5μm. **(B)** The protein levels of IFI16 and HuR after overexpression of ARPC1B in GSC 8-11. **(C)** The mRNA expression of IFI16 and HuR assessed by qRT-PCR assay. **(D)** The protein levels of IFI16 and HuR in shNC or shARPC1B-GSCs treated with 100μg/ml CHX for indicated times. **(E)** Representative images and quantification of IHC staining for ARPC1B, HuR and IFI16 in different groups of GSC 267 xenograft sections (scale bar, 200μm).


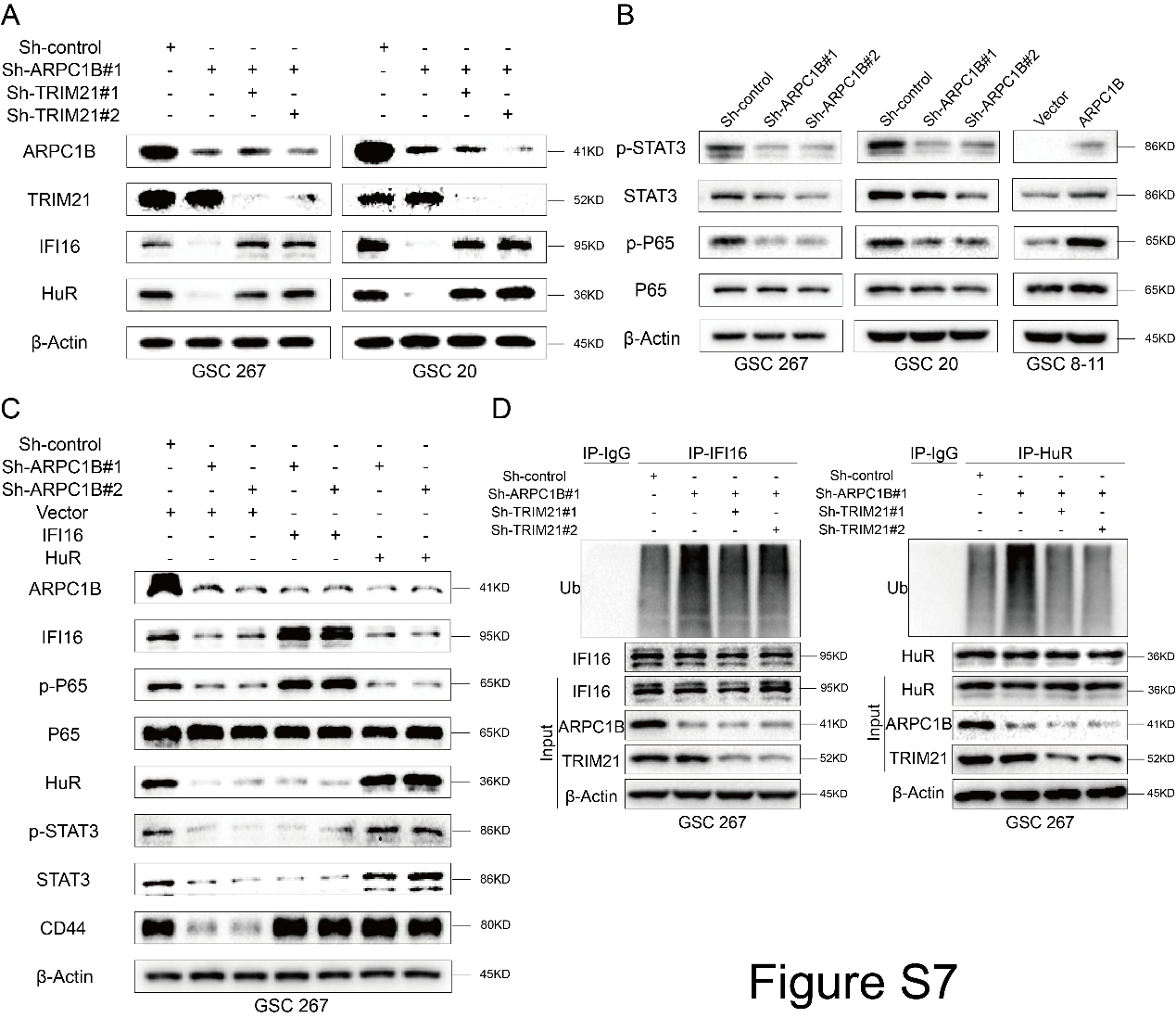


**Fig. S7 (A)** Western blotting analysis showing the effect of TRIM21 knockdown on the protein levels of IFI16 and HuR in sh-ARPC1B GSCs. **(B)** Western blotting analysis showing the effect of ARPC1B knockdown in GSC 20 and GSC 267, or ARPC1B overexpression in GSC 8-11 on the protein levels of STAT3, p-STAT3, P65, and p-P65. **(C)** Western blotting analysis of protein levels of ARPC1B, IFI16, HuR, P65, p-P65, STAT3, p-STAT3 and, CD44 in GSC 20 treated with indicated interventions. **(D)** Western blotting analysis showing that knockdown of TRIM21 could reverse the effect of ARPC1B inhibition on IFI16 and HuR ubiquitination. GSCs were pretreated with MG132 (10 µM) for 6 hours before cell lysates were collected.


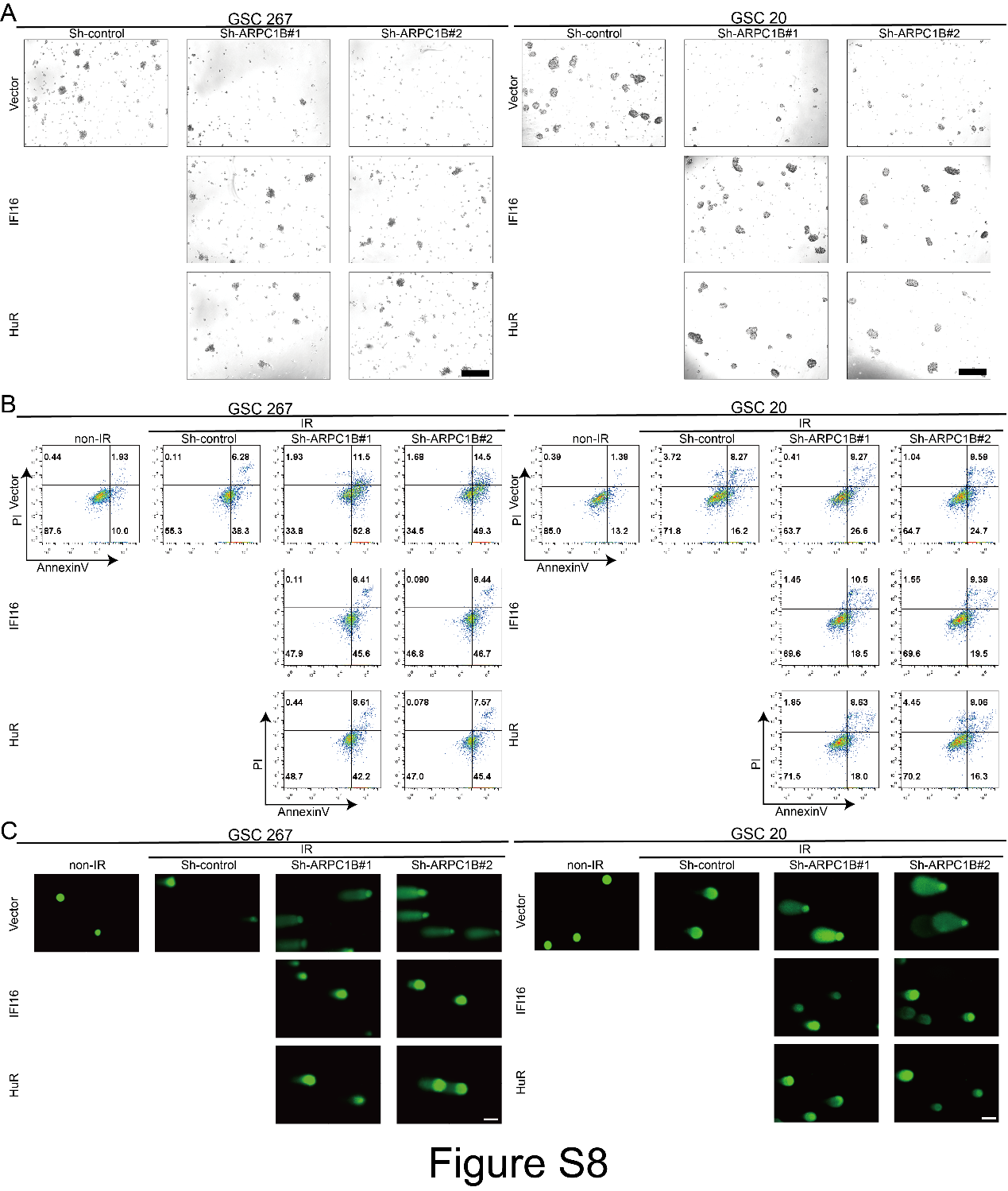


**Fig. S8 (A)** Representative images of tumor sphere formation of GSC 267 and GSC 20 treated with indicated interventions. Scale bar, 100μm. **(B)** Representative images of flow cytometry assays showing apoptosis of GSC 267 and GSC 20 treated with indicated interventions. **(C)** Representative images of comet assays showing DNA damage of GSC 267 and GSC 20 treated with indicated interventions. Scale bar, 20μm.


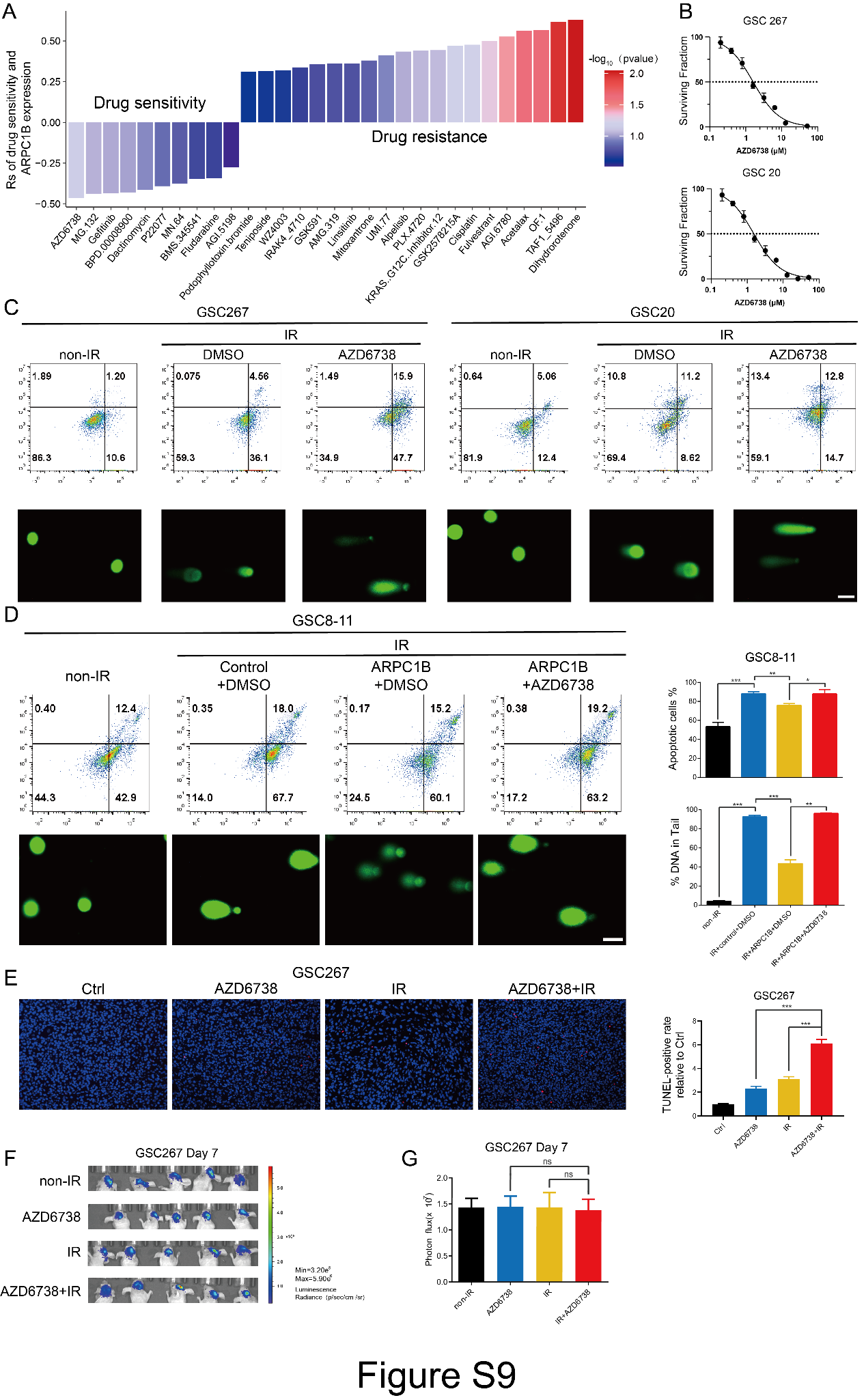


**Fig. S9 (A)** The correlation between the ARPC1B expression and drug sensitivity assessed by Spearman algorithm. **(B)** CCK-8 assay in GSC 267 and GSC 20 treated with different concentrations of AZD6738 for 48 h. **(C)** Representative images and quantification of apoptosis assays (upper panel) and comet assays (lower panel, scale bar, 20μm) for GSC 20 and GSC 267 upon treatment with the indicated interventions. **(D)** The representative images and quantification of apoptosis assays (upper panel) and comet assays (lower panel, scale bar, 20μm) for GSC 8-11 upon treatment with the indicated interventions. The right panels are the quantification of apoptosis rate and DNA damage, respectively. **(E)** Representative images and quantification of TUNEL staining in sections of GSC 267 xenografts for different groups. Scale bar, 200μm. **(F)** Bioluminescence imaging of tumor size on day 7 in GSC 267 xenograft nude mice treated with the indicated interventions. **(G)** The quantification of photon counts on day 7 of the GSC 267 xenografts.

**Supplementary Materials and Methods**

**Antibodies used**

For western blotting, the following antibodies were used in this study:

β-actin (Cell Signaling Technology, 8480), ARPC1B (Santa cruz, 137125), IFI16 (Cell Signaling Technology, 14970), HuR (Cell Signaling Technology, 12582), HA-tag (Cell Signaling Technology, 3724), Flag-tag (Cell Signaling Technology, 8146), TRIM21 (Santa cruz, 25351), P65 (Cell Signaling Technology, 8242), p-P65 (Cell Signaling Technology, 3033), STAT3 (Cell Signaling Technology, 9139), p-STAT3 (Cell Signaling Technology, 4113), DBN1 (Santa cruz, 374269), ACTN4 (Santa cruz, 17829), FLNA (Santa cruz, 17749), CORO1C (Santa cruz, 376919), CD44 (Cell Signaling Technology, 3570), YKL-40 (Cell Signaling Technology, 47066), SOX2 (Cell Signaling Technology, 3579), γ-H2AX (Cell Signaling Technology, 7631), Ubiquitin (Cell Signaling Technology, 3933), ATR (Santa cruz, 515173).

The following antibodies were used in IF assay:

ARPC1B (Santa cruz, 137125), γ-H2AX (Cell Signaling Technology, 7631), HuR (Cell Signaling Technology, 12582), IFI16 (abcam, 169788).

CD44 (Cell Signaling Technology, 3570), IFI16 (Cell Signaling Technology, 14970) and HuR (Santa cruz, 5261) used for IHC assay.

**Primers used for qRT-PCR were listed**

Human β-actin:

5’CATGTACGTTGCTATCCAGGC3’

5’CTCCTTAATGTCACGCACGAT3’

Human HuR:

5′-GCTCGGTCTACTCAGGCATC-3′

5′-CCAGTCCAGGAGCCTAATGA-3′

Human IFI16:

5’TAGAAGTGCCAGCGTAACTCC3’

5’TGATTGTGGTCAGTCGTCCAT3’

**RNA interfering sequence**

sh-Control:

UUCUCCGAACGUGUCACGUTT

ACGUGACACGUUCGGAGAATT

sh-ARPC1B#1

GCUCUCGUGUGAUCUCCAUTT

AUGGAGAUCACACGAGAGCTT

sh-ARPC1B#2

GGGUACAUGGCGUCUGUUUTT

AAACAGACGCCAUGUACCCTT
